# Dietary Intake Among Community-Dwelling Adults Aged 55 Years and Older in the Central Division, Fiji

**DOI:** 10.3390/nu18142354

**Published:** 2026-07-17

**Authors:** Salanieta Naliva, Marlena C. Kruger, Palatasa Havea, Gade Waqa, Jacqui Webster, Briar L. McKenzie, Colin Bell, Carol A. Wham

**Affiliations:** 1 School of Sport, Exercise & Nutrition, College of Health, Massey University, Albany, Auckland 0632, New Zealand; c.a.wham@massey.ac.nz; 2 Riddet Institute, Massey University, Palmerston North 4442, New Zealand; m.c.kruger@massey.ac.nz (M.C.K.); p.havea@massey.ac.nz (P.H.); 3 School of Health Sciences, College of Health, Massey University, Tennent Drive, Palmerston North 4410, New Zealand; 4 Pacific Student Success, Massey University, Tennent Drive, Palmerston North 4410, New Zealand; 5 C-POND, Fiji Institute of Pacific Health Research, Fiji National University, Suva, Fiji; gade.waqa@fnu.ac.fj; 6 WHO Collaborating Centre Nursing, Midwifery and Health Development, University of Technology, Sydney, NSW 2007, Australia; jacqui.webster@uts.edu.au; 7 The George Institute for Global Health, University of New South Wales (UNSW), Sydney, NSW 2031, Australia; bmckenzie@georgeinstitute.org.au; 8 Institute for Health Transformation, Deakin University, Geelong, VIC 3220, Australia; colin.bell@deakin.edu.au

**Keywords:** dietary intake, nutrition, ageing, older adults, Fijians, protein

## Abstract

Background/Objectives: This study aimed to describe dietary intake among adults 55 years and older living in the Central Division of Fiji and identify food sources of energy, macronutrients, and micronutrients. Methods: A retrospective dietary dataset consisting of 171 adults aged 55 and over was collected using the 24 h multiple-pass recall (24 h MPR) and assessed using the Intake24 Fiji smartphone application. Analyses were restricted to 147 participants with complete dietary record data. Energy, macronutrient, and selected micronutrient intakes were assessed against recommended amounts, and nutrient sources were reported for 15 food groups. Results: Mean daily energy intakes (6.6 MJ/day) were lower than recommended, especially for Those > 70 years (5.3 MJ). The participants had acceptable contributions of energy from fat (28%) and carbohydrates (56%) but lower contributions for protein (13%) than the recommended range (15–25%). The mean protein intake for men and women was 0.68 g/kg/day. More than half of these older adults consumed less than the Estimated Average Requirement (EAR) of retinol, thiamin, folate, vitamin C, vitamin E, calcium, iron, manganese, selenium, and zinc, while vitamin B_6_ requirements were met by approximately half of the participants. The primary sources of energy and nutrients were from bread and bakery products, mixed dishes, and sugar-sweetened beverages. Conclusions: The dietary intakes of older adults in Fiji’s Central Division met carbohydrate and fat intake guidelines but were low in protein as well as retinol, thiamin, folate, vitamin C, vitamin E, and minerals such as calcium, iron, manganese, selenium, and zinc; however, approximately half of the participants met the vitamin B6 requirements. These findings highlight the need to improve overall diet quality for older Fijians by increasing the accessibility of nutrient-dense, protein-rich foods and addressing low energy and micronutrient intakes.

## 1. Introduction

In Fiji, the proportion of adults aged 55 years and above is currently 14%, and it is projected to increase to 17% by 2050, with more than 50% comprising Fijians of iTaukei (indigenous) descent [1,2]. This shift is occurring alongside a high burden of diet-related non-communicable diseases (NCDs), namely, cardiovascular diseases (CVDs), including heart attack, angina (chest pain related to heart disease), and stroke, which are the leading causes of premature mortality in the country. Approximately 80% of all deaths in Fiji are attributed to NCDs, and 31% of these deaths occur before age 70. This proportion continues to rise [3]. Circulatory system diseases were responsible for the highest age-standardised mortality rates in both older men and women. In comparison, among women aged 75+ (but not older men), there was a significant annual increase in mortality rates from endocrine, nutritional, and metabolic diseases [4].

In upper-middle-income countries such as Fiji, shifting dietary patterns, together with population ageing, may be contributing to stagnation in Life Expectancy (LE). This transition from traditional, nutrient-dense foods towards more processed and energy-dense dietary patterns may exacerbate nutrient deficiencies and chronic disease risk among older Fijians [4,5,6,7]. National health and nutrition surveillance systems in Fiji have prioritised child and maternal nutrition [5], overlooking the need for age-appropriate dietary policy and public health programming. Fiji has National Food and Health Guidelines aimed at the general population, including broad messages about choosing a variety of local foods and promoting healthy lifestyles [8]. Similarly, the Pacific region endorses wider healthy-living guidelines through the Secretariat of the Pacific Community (SPC), which emphasises balanced diets from core food groups and the use of local foods [9]. There are currently no Pacific- or Fiji-specific nutrient reference values (NRVs) with which to guide the assessment of adequate nutrient intake. In the absence of local guidelines, the Australian and New Zealand nutrient reference values (NHMRC) are commonly applied in Fiji, as they provide the most relevant and regionally aligned standards for evaluating dietary adequacy in similar population contexts [10,11]. Furthermore, the Pacific guidelines mentioned earlier do not offer specific guidance for older adults, underscoring a critical gap in nutrition policy.

Nutrition is a cornerstone of successful ageing, as food not only is critical for functional independence but also impacts the overall quality of life in older adults globally. The ageing process affects nutrient needs such that requirements for energy may be reduced while requirements for other nutrients, such as protein and calcium, may be increased. The guiding value for energy intake in older persons is 30 kcal/kg of bodyweight (BW)/day [12] or its equivalent, 0.13 MJ/kg BW/day. It is further suggested that energy intake should be individually adjusted regarding nutritional status, physical activity, disease status, and tolerance. In Australia and New Zealand, the current recommended daily intake of protein has been established as 0.75 g/kg BW/day for females and 0.84 g/kg BW/day for males aged 51–70 years, whereas it is 0.94 g/kg bodyweight (BW)/day for females and 1.07 g/kg BW/day for males aged 70 years or older [10].

However, growing evidence suggests that older adults may require higher amounts of protein for maintenance of lean body mass, body functions, and health. Daily amounts of 1.0–1.2 g/kg BW have been suggested for healthy older people. In case of illness, protein requirements may further increase because of inflammation, infections, and wounds. A further 1.2–1.5 g/kg BW has been advised for older persons with chronic conditions and up to 2.0 g/kg BW/day in the case of severe illness, injury, or malnutrition [12]. In addition, to support muscle protein synthesis, protein intake should be evenly distributed across meals, with approximately 25–30 g per meal [13]. The Estimated Average Requirement (EAR) for calcium increases from 840 mg/day for adults aged 51–70 years to 1100 mg/day for men aged >70 years; for women, the EAR is 1100 mg/day from 51 years of age onwards [10]. Older people require less energy intake than younger people because of age-related physiological changes, including a decreased basal metabolic rate (BMR) and reduced lean body mass. Lower levels of physical activity and engagement in fewer physically demanding activities further lower older adults’ total energy expenditure (TEE) [14,15,16]. Despite their lower energy needs, older people still require adequate energy and nutrient intakes [17] to support their health. Adequate protein intake is especially important for older adults to reduce age-related inflammation [18] and prevent muscle loss [19,20]. However, protein intake may often be inadequate in older populations [21,22,23]. Furthermore, adequate micronutrient intake becomes increasingly important with ageing due to its role in immune function, bone health, and reducing age-related decline. Thus, as people age, their macronutrient and micronutrient needs change owing to changes in metabolism and energy expenditure.

The 2004 Fiji National Nutrition Survey (NNS) surveyed individuals 15 years of age and older and found that two-thirds (60.8%) of the adult population met the United States Dietary Reference Intake (USDRI) for protein (50 g/day) [24]. Adults aged ≥18 years in the 2015 Fiji National Nutrition Survey had a reported mean daily protein intake of 149 g, exceeding the recommended dietary intake (RDI) of 64 g/day. At the same time, the micronutrient intakes of several key vitamins and minerals fell short of the Australia/New Zealand nutrient reference values (NRVs), except for sodium, for which roughly one-quarter to one-third of the participants were below the recommended levels [11]. In both the Fiji National Nutrition surveys 2004–2015, data for those above 18 years of age were aggregated; therefore, the dietary intake and nutritional adequacy of adults ≥ 55 years living in communities in Fiji are unknown. Furthermore, socioeconomic factors, including poverty and income disparities, play a critical role in shaping dietary intake and nutritional outcomes. In Fiji, marked regional inequalities exist, with significantly higher poverty rates reported in rural areas than in urban areas of the Central Division (40.6% vs. 17.9%) [25]. Such disparities may influence food access, affordability, and dietary choices, potentially contributing to observed differences in nutrient intake between regions. The aim of this study was to describe the dietary intakes of adults 55 years and older living in the Central Division of Fiji and identify food sources of energy, macronutrients (particularly protein), and micronutrients.

## 2. Materials and Methods

### 2.1. Study Population

A retrospective study was undertaken between March and July 2022 to analyse the dietary intakes of adults 55 years and over via data collected from a previous study conducted in the Central Division of Fiji in 2022 [26,27,28,29]. In the latter study, a 24 h multiple-pass recall (24 h MPR) dietary assessment method was applied as part of the broader Global Alliance for Chronic Diseases (GACD) project to investigate food intake among adults aged 18 to 85 years [27]. The total number of participants was 530, of whom 171 were ≥55 years and therefore included in this study.

### 2.2. Sample Size and Recruitment

Sampling was based on obtaining a representative sample of adults aged 18 years and older, as described previously [26,27,28,29].

### 2.3. Study Measures and Variables

- The protocol for the diet survey is described as part of a broader project conducted to investigate the food environment in Fiji [27]. Research assistants collected demographic and health data of the participants using a smartphone data collection app. This entailed a general questionnaire on demographic information, disease history, and medication. The study measures and variables for the health and socio-demographic data, chronic diseases, and anthropometric data are described in the study methods [26,28].
- Twenty-four-hour multiple-pass recall (24 h MPR) was employed.

A 24 h MPR was conducted, requiring participants to recall all foods and drinks they had consumed in the previous 24 h. This was facilitated by the interviewer-administered use of Intake24, which is an app-based 24 h diet recall software product that allows for the entry of foods consumed and automatically converts input information into macronutrient and micronutrient intake data [30]. For the survey in Fiji, the Intake24 application was adapted to include commonly consumed foods in Fiji, as previously described [26,28]. 

### 2.4. Ethical Approval

Ethical approval for the collection of these data was provided by the University of New South Wales (#HC200469) and the Fiji National University Human Health Research Ethics Committee (HHREC 264.20). The process of gaining access to the participants and their data was also overseen by FNU and approved by the HHREC. Ethical approval for this study was obtained from both the Massey University Human Ethics Committee (MUHEC) Ethics Application—OM1 23/34 and the Fiji National University Human Health Research Ethics Committee (FNU-HHREC) under Number 24/2023.

### 2.5. Current Study: Dietary Intakes of Older Adults (≥55 Years and Older)

#### 2.5.1. Study Measures and Variables

- Inclusion criteria

We included participants aged >55 years and above residing in the selected study sites (namely, the rural area Deuba and the urban area Waidamudamu) who were physically and cognitively able to provide informed consent and participate in the survey.

Exclusion criteria

Participants below the age of 55 and those with inadequate or missing 24 h MPR records were excluded.

ii. Health and socio-demographic data included age (≥55 years); sex (female or male); ethnicity (whether one is a Fijian of iTaukei, Indian, or Other descent (FID/FOD)); whether one lives in a rural (Deuba) or urban (Waidamudamu) area; education (having achieved a secondary-level education or below) and tertiary education (university); household type (living alone or in a shared household); self-assessed health, which was evaluated using a single-item measure with response categories (excellent, very good, good, fair, or poor); medication use (yes or no); chronic diseases (high blood pressure, low blood pressure, high cholesterol, heart attacks, stroke, angina, and diabetes); and systolic blood pressure, diastolic blood pressure, and hypertension (classified as systolic blood pressure ≥ 140 mmHg or diastolic blood pressure ≥ 90 mmHg). Three blood pressure readings were taken according to the WHO STEPS standard procedure [31]. Blood pressure was measured using a digital automatic blood pressure monitor (OMRON M6). Living with hypertension was classified as a systolic blood pressure ≥ 140 mmHg, diastolic blood pressure ≥ 90 mmHg, or self-reported use of antihypertensive medications. During data analysis, the mean of the second and third readings was calculated.
iii. Anthropometric measurements
  We collected height (m), weight (kg), waist circumference, and body mass index (BMI) using the World Health Organization (WHO) BMI classification: underweight (<18.5 kg/m^2^); normal weight (18.5 to 24.9 kg/m^2^); overweight (25.0 to 29.9 kg/m^2^); and obese (≥30.0 kg/m^2^) [32]. We used the updated BMI classifications for older adults: <24 kg/m^2^ (underweight), 24–29 kg/m^2^ (normal), and >29 kg/m^2^ (excess body weight) [29,30]. Evidence suggests the recommended WHO guidelines are appropriate for younger and middle-aged adults; however, a higher BMI range is recommended for older adults based on functional capabilities and other comorbidities [33]. BMI was analysed as both a continuous variable (mean ± SD) and as categorical classifications presented as proportions according to standard cut-offs.
iv. Nutrient intake from the 24 h MPR application—Intake24

We collected information on total energy (MJ) and macronutrients (g)—carbohydrates, protein, and fat as well as total sugars, sucrose, and fatty acids (SFAs)—and micronutrients (in mg, except where specified), namely, retinol, thiamin, vitamin B6, vitamin B12, folate, vitamin C, vitamin E, calcium, iron, magnesium, phosphorus, potassium, manganese, selenium, copper, and zinc. See Supplementary File, Table S1.

v. Food sources

We reported food sources in 15 categories used in previous studies [33,34]: 1—bread and bakery products; 2—cereal and grain products; 3—coconut products; 4—convenience foods (including takeaway meals, ready meals, meal kits, and pre-prepared salads and sandwiches); 5—dairy; 6—edible oil and oil emulsions; 7—egg and egg products; 8—fruit, vegetables, nuts, and legumes; 9—meat, poultry, and meat alternatives; 10—mixed cooked dishes; 11—non-alcoholic beverages; 12—sauces, dressings, spreads, and dips; 13—seafood and seafood products; 14—snack foods (sweet and savoury snacks); and 15—table sugars, honey, and related products (such as syrups and molasses). See Supplementary File, Table S2.

#### 2.5.2. Data Processing

Energy and macronutrient intakes were calculated as both kilocalories and kilojoules per day for energy; grams per day for fat, protein, carbohydrate, saturated fatty acids (SFAs), total sugars, and sucrose; and grams per kilogram of bodyweight per day for protein. Carbohydrate, fat, protein, SFA, and sugar intakes were also reported as percentages of total energy intake [26]. 

#### 2.5.3. Data Analysis

Complete case analyses were conducted after 24 participants (14%) were removed from the analysis because of missing or implausible data. One participant was removed as an outlier for protein intake, while 23 participants consented to participate in other aspects of the survey, but not the dietary recall, or other reasons were given, hence the difference in numbers. Only 147 participants’ dietary intakes were analysed as reflected in Table 2, Table 3, Table 4, Table 5, Table 6 and Table 7. Analyses were restricted to participants with complete dietary records. Analyses were weighted to reflect the probability of individual selection (sample weight) and match the population structure of Deuba and Waidamudamu (population weight). Weights were based on sex (female or male), age group (55–69 and >70), site (urban or rural), ethnicity (iTaukei (Indigenous Fijian), Fijian of Indian descent, or other (FID/FOD)), and the reported population structure of the Central Divisions, as used previously [26,28]. Statistical analysis was performed using SPSS version 29.0 (IBM Corp, USA). The age groups were stratified into two groups, 51–69 years and >70 years, according to the nutrient reference value (NRV) grouping for older adults [10]. In the absence of Pacific acceptable ranges, a description of total energy, macronutrients, acceptable macronutrient distribution range (AMDR), micronutrient intake, and the food sources of the macronutrients and micronutrients included above was analysed via the 24 h MPR using Intake24 Fiji software.

Dietary intake data were evaluated against the Estimated Average Requirement (EAR) [10]. The EAR represents the intake level estimated to meet the requirements of 50% of individuals in a specific life stage and sex group. It is used to assess nutrient adequacy at the population level. Intakes below the EAR were interpreted as indicating a higher likelihood of inadequacy. Meeting or exceeding the EAR was not considered sufficient to indicate adequate intake for individuals, as the EAR reflects only the median requirement.

Descriptive statistics were generated for participants’ characteristics and dietary intake data (means and medians). Continuous data were assessed for normality using visual inspection of histograms and Q–Q plots. Non-normally distributed variables were analysed using the Mann–Whitney U test. The results were reported as means ± standard deviations (SDs) for continuous variables if normally distributed (this mostly applied for the macronutrients) and medians and interquartile ranges (25th and 75th percentiles) if non-normally distributed (micronutrients) and proportions for categorical variables with 95% confidence intervals (CIs). Differences between groups were analysed using Mann–Whitney U tests for non-normally distributed scale data and a chi-squared test of independence for categorical data. Dietary adequacy was assessed using the proportion of participants below EARs and within AMDRs, as recommended for population-level nutritional assessment; mean and median values were used for descriptive purposes only. These NRVs are considered the most regionally relevant and provide a consistent benchmark for comparing dietary adequacy in older Fijian populations [10]. Food sources were compared to those reported previously in the 2015 NNS [11] and papers [26,28,29]. Given that dietary data were collected using a single 24 h recall, the micronutrient intake estimates are exploratory. They should be interpreted with caution, as they do not capture usual intake or intra-individual variability. Results were considered significant at *p* < 0.05.

## 3. Results

### 3.1. Health and Sociodemographic Characteristics

A total of 147 older adults were included in our analyses (response rate: 86%), of whom 59% were women, with a mean age of 64 (95% CI, 62.9–65.1) years. Eighty-one percent of the participants were 55–70 years of age at the time of the survey, and two-thirds were of FID/FOD. Eighty percent had attained secondary education or lower, and most people lived in a household with others (Table 1). Two-thirds of the participants reported their health was either good, very good, or excellent, and over a quarter (29%) reported taking medications regularly. Forty percent reported they had been diagnosed with high blood pressure (43.3%) (35.7–51.1). The mean systolic blood pressure of the participants was 150.2 (146.5–153.8) mmHg, the mean diastolic blood pressure was 88.3 (86.1–90.5) mmHg, and 71.6% (63.6–78.7) of the participants had hypertension (systolic blood pressure ≥ 140 mmHg or diastolic blood pressure ≥ 90 mmHg).

According to the WHO Classification of BMI for adults, a quarter (27%) of the participants were overweight (BMI > 25.0 to 29.9 kg/m^2^), corresponding to more men (37.3%) than women (19.3%). Twelve percent of the participants were living with obesity (BMI ≥ 30.0 kg/m^2^), with this group consisting of more women (17%) than men (5%). Using Winter et al.’s cut-points for older adults (>65 years), applied across the ≥55-year sample for comparative purposes, we classified 54.2% of the participants as underweight, with a higher proportion among women than men (56.4% vs. 50.9%). Additionally, 28.7% were classified as normal weight and 17.1% as overweight or obese. However, these estimates should be interpreted with caution, as the majority of the participants were 55–69 years old and the cut-points are designed for adults > 65 years [33,34].

**Table 1:** Health and socio-demographic characteristics of the participants (N = 147).

Variables	Weighted Estimates	Women N = 78	Men N = 69
**Gender (%, 95% CI)**			
Women	59.1(51.3–66.5)		
Men	40.9 (33.5–48.7)		
Age (mean, 95%CI)	64.0 (62.9–65.1)	65.0 (63.3–66.7)	62.9 (61.5–64.4)
**Age group (%, 95% CI)**			
55–69 years	81.3 (74.6–86.8)	76.2 (66.6–84.1)	88.6 (78.7–94.9)
>70 years	18.7 (13.2–25.4)	23.8 (15.9–33.3)	11.4 (5.1–21.3)
**Ethnicity (%, 95% CI)**			
iTaukei	37.4 (30.2–45.1)	38.6 (29.1–48.8)	35.7 (24.6–48.1)
Fijians of Indian descent and others	62.6 (54.9–69.8)	61.4 (51.2–70.9)	64.3 (51.9–75.4)
**Area (%, 95% CI)**			
Deuba	50.3 (42.6–58.0)	50.5 (40.4–60.6)	50.0 (37.8–62.2)
Waidamudamu	49.7 (42.0–57.4)	49.5 (39.4–59.6)	50.0 (37.8–62.2)
**Education ^¥^ (%, 95% CI)**			
Secondary education or below	81.3 (74.6–86.8)	85.1(76.7–91.4)	75.7 (64.0–85.2)
Tertiary education (university)	5.3 (2.4–9.8)	3.0 (0.6–8.4)	8.6 (3.2–17.7)
**Household Type ^¥^ (%, 95% CI)**			
Living alone	5.8 (2.8–10.5)	7.9 (3.5(15.0)	2.9 (0.3–9.9)
Shared household	80.7 (74.0–86.3)	80.2 (71.1–87.5)	81.4 (70.3–89.7)
**Self-assessed health ^¥^ (%, 95% CI)**			
Excellent	6.4 (3.3–11.2)	5.9 (2.2–12.5)	7.1 (2.4–15.9)
Very good	24.6 (18.3–31.7)	23.8 (15.9–33.3)	25.7 (16.0–37.6)
Good	36.3 (29.1–43.9)	32.7 (23.7–42.7)	41.4 (29.8–53.8)
Fair	15.8 (10.7–22.1)	19.8 (12.5–28.9)	10.0 (4.1–19.5)
Poor	3.5 (1.3–7.5)	5.9 (2.2–12.5)	0.0 (0.0–0.51)
**Taking medication regularly ^¥^ (Yes %, 95% CI)**			
Yes	29.2 (22.5–36.7)	32.7 (23.7–42.7)	24.3 (14.8–36.0)
No	57.3 (49.5–64.8)	55.4 (45.2–65.3)	60.0 (47.6–71.5)
**Chronic diseases ^¥^ (Yes %, 95% CI)**			
High blood pressure, mmHg	43.3 (35.7–51.1)	47.5 (37.5–57.7)	37.1 (25.9–49.5)
Low blood pressure, mmHg	6.4 (3.3–11.2)	9.9 (4.9–17.5)	1.4 (0.0–7.7)
High cholesterol	11.7 (7.3–17.5)	11.9 (6.3–19.8)	11.4 (5.1–21.3)
Heart attack	4.1 (1.7–8.3)	4.0 (1.1–9.8)	4.3 (0.9–1.2)
Stroke	1.2 (0.1–4.2)	2.0 (0.2–7.0)	0.0 (0.0–5.1)
Angina	11.7 (7.3–17.5)	16.8 (10.1–25.6)	4.3 (0.9–1.2)
Diabetes	22.2 (16.2–29.2)	23.8 (15.9–33.3)	20.0 (11.4–31.3)
Systolic blood pressure, mmHg (mean, 95% CI)	150.2 (146.5–153.8)	155.2 (149.5–160.8)	144.5 (140.3–148.6)
Diastolic blood pressure, mmHg (mean, 95% CI)	88.3 (86.1–90.5)	89.1 (85.8–92.3)	87.3 (84.3–90.3)
**Hypertension ^¥ a^ (n, 95% CI)**			
Normal blood pressure	28.4 (21.3–36.4)	28.1 (19.1–38.6)	28.8 (17.8–42.1)
Hypertension	71.6 (63.6–78.7)	71.9 (61.4–80.9)	71.2 (57.9–82.2)
**Anthropometric measurements**			
Height, m (mean, 95% CI)	1.62 (1.59–1.64)	1.56 (1.53–1.58)	1.68 (1.65–1.72)
Weight, kg (mean, 95% CI)	78.7 (75.3–82.0)	75.1 (70.8–79.4)	82.7 (77.6–87.9)
Waist circumference, cm (mean, 95% CI)	103.95 (98.8–109.1)	101.9 (97.8–105.9)	106.3 (96.2–116.4)
BMI ^b^, kg/m^2^ (mean, 95% CI)	24.5 (23.1–25.9)	24.1 (22.6–25.5)	25.1 (22.6–27.5)
***BMI classification* ^c,d^** **(%, 95% CI)**			
Underweight	15.6 (10.2–22.5)	17.0 (9.9–26.6)	13.6 (6.0–25.0)
Normal	45.6 (37.4–54.0)	46.6 (35.9–57.5)	44.1 (31.2–57.6)
Overweight	26.5 (19.6–34.4)	19.3 (11.7–29.1)	37.3 (25.0–50.9)
Obese	12.2 (7.4–18.7)	17.0 (9.9–26.6)	5.1 (1.1–14.1)
***BMI classification* ^d,e^** **(%, 95% CI)**			
Underweight	54.2 (41.8–68.2)	56.4 (40.3–75.3)	50.9 (32.4–73.7)
Normal	28.7(21.8–36.4)	23.1(15.1–32.6)	37.1 (25.2–50.4)
Excess BW	17.1(11.0–25.0)	20.5 (12.0–31.9)	12.0 (5.7–23.4)

^¥^ missing values, N = 23 (F = 12, M = 11). ^a^ Hypertension is classified as systolic blood pressure ≥ 140 mmHg or diastolic blood pressure ≥ 90 mmHg. ^b^ Body mass index. ^c^ WHO BMI classification: *underweight* (<18.5 kg/m^2^); *normal weight* (18.5 to 24.9 kg/m^2^); *overweight* (25.0 to 29.9 kg/m^2^); *obese* (≥30.0 kg/m^2^) [32]. ^d^ Missing value, N = 24 (F = 12, M = 12) ^e^ BMI classification for >65-year-olds: underweight (<24 kg/m^2^); normal weight (24 to 29 kg/m^2^); excess body weight-BW [Overweight and Obese] (>29 kg/m^2^) [34,35].

### 3.2. Energy Daily Macronutrient Intake According to Sex, Age, Ethnicity, and Locality

The mean (standard deviation) energy intake was 6.6 (3.1) megajoules per day (MJ/d), with men and women consuming 6.9 MJ/day and 6.3 MJ/day, respectively. These values are lower than the Estimated Energy Requirements (EERs) for men (9.3–11.7 MJ/day) and women (8.3–9.6 MJ/day) [10]. The mean macronutrient distribution range for protein was 13.0 ± 5.8% of total energy intake; fat contributed 28.3 ±12.8%; and carbohydrates accounted for 56.2 ± 14.3%. The mean protein contribution to energy for younger participants was 13.4 ± 6.13% vs. 11.70 ± 4.25% for Those > 70 years. Protein contribution to total energy intake was 13.5 ±6.2% for women and 12.5± 5.4% for men, with a similar fat contribution to energy for women (28.8 ± 12.8%) and men (27.7 ± 12.7%), while the carbohydrate contribution for women was 55.3 ± 14.2%, and that for men was 57.2 ± 14.6. Those > 70 years old consumed a higher % of energy from carbohydrates relative to the younger participants (62.9 vs. 54.7%, *p* = 0.007). Fijians of Indian/other descent had higher fat and lower carbohydrate intakes than iTaukei Fijians: fat, 31.4 vs. 24.0% (*p* = 0.014); carbohydrates, 53.4 vs. 60.2 (*p* = 0.007) (Table 2). The mean protein intake was 0.68 g/kg/day. Protein intake differed significantly by age group (55–69 years vs. ≥70 years, *p* = 0.040), with higher intake observed among those aged 55–69; however, no significant differences were found according to ethnicity or rural-versus-urban residence (*p* > 0.05 for these comparisons; see Table 2).

**Table 2:** Mean daily macronutrient intake by sex, age, ethnicity, and locality (N = 147).

	All Participants N = 147	By Gender		By Age Groups		By Ethnicity		By Locality		
		Women (n = 78)	Men (n = 69)	55–69 Years (n = 129)	>70 Years (n = 18)	iTaukei (n = 61)	FID&FOD (n = 86)	Deuba (Rural) (n = 81)	Waidamudamu (Urban) (n = 66)	
Nutrients		Mean ±SD								*p* (Age Groups) ^†^
Energy (kcal)	1560.96 ± 730.55	1495.16 ± 727.62	1635.06 ± 732.01	1631.19 ± 707.54	1247.13 ± 762.51	1440.45 ± 753.65	1646.10 ± 705.80	1571.63 ± 698.11	1547.88 ± 773.66	0.013
Energy (MJ)/(kJ)	6569.95 ± 3067.72	6292.77 ± 3056.14	6882.12 ± 3072.84	6862.72 ± 2968.23	5261.68 ± 3219.17	6074.79 ± 3172.94	6919.81 ± 2959.83	6615.96 ± 2931.44	6513.56 ± 3248.76	0.014
Fat (g)	53.35 ± 37.89	51.95 ± 38.82	54.91 ± 37.04	57.83 ± 38.90	33.35 ± 25.02	41.03 ± 33.74	62.05 ± 38.43	53.37 ± 36.94	53.31 ± 39.31	<0.001
Fat (% energy)	28.33 ± 12.75	28.84 ± 12.82	27.74 ± 12.75	29.54 ± 12.72	22.91 ± 11.60	23.97 ± 14.40	31.40 ± 10.49	27.73 ± 12.50	29.05 ± 13.11	0.014
Protein (g)	51.07 ± 42.12	49.58 ± 50.07	52.74 ± 31.12	54.45 ± 44.48	35.98 ± 24.80	50.90 ± 58.82	51.19 ± 24.71	54.67 ± 51.91	46.65 ± 25.19	0.040
Protein (g/kg)	0.68 ± 0.51	0.68 ± 0.57	0.68 ± 0.44	0.72 ± 0.53	0.52 ± 0.38	0.58 ± 0.65	0.76 ± 0.38	0.71 ± 0.60	0.65 ± 0.37	0.063
Protein (% energy)	13.06 ± 5.86	13.53 ± 6.22	12.54 ± 5.42	13.37 ± 6.13	11.70 ± 4.25	13.29 ± 7.48	12.91 ± 4.41	13.53 ± 7.19	12.50 ± 3.59	0.183
Carbohydrate (g)	223.72 ± 106.24	211.72 ± 106.29	237.22 ± 105.33	227.70 ± 100.13	205.93 ± 130.89	221.86 ± 115.65	225.03 ± 99.75	223.16 ± 102.04	224.41 ± 111.97	0.423
Carbohydrate (% energy)	56.23 ± 14.39	55.32 ± 14.21	57.26 ± 14.63	54.73 ± 14.19	62.99 ± 13.58	60.21 ± 17.50	53.42 ± 10.98	56.41 ± 15.57	56.02 ± 12.91	0.007
Saturated FA (g)	21.88 ± 15.83	20.48 ± 15.83	23.46 ± 15.80	23.73 ± 16.29	13.65 ± 10.29	15.96 ± 14.26	26.07 ± 15.61	20.89 ± 15.38	23.10 ± 16.40	<0.001
SFA (% energy)	11.82 ± 7.09	11.51 ± 6.53	12.16 ± 7.69	12.32 ± 7.28	9.57 ± 5.73	9.67 ± 8.57	13.33 ± 5.37	10.94 ± 6.39	12.89 ± 7.77	0.069
Total sugar (g)	63.49 ± 50.87	58.23 ± 51.30	69.42 ± 50.09	64.83 ± 50.68	57.50 ± 52.24	71.16 ± 62.96	58.08 ± 39.73	68.63 ± 58.78	57.20 ± 38.58	0.501
Sugar (% energy)	15.73 ± 11.22	14.65 ± 11.23	16.94 ±11.16	15.57 ± 11.40	16.41 ± 10.57	18.71 ± 13.50	13.62 ± 8.77	16.91 ± 12.96	14.28 ± 8.49	0.714
Sucrose	35.12 ± 35.15	31.54 ± 35.21	39.15 ± 34.89	34.91 ± 35.11	36.07 ± 35.99	37.94 ± 43.23	33.12 ± 28.19	36.93 ± 39.74	32.90 ± 28.68	0.878

^†^ Comparing all age groups, 55–69 years, and >70 years, *p* < 0.05, independent-samples *t*-test. *p*-values are presented for comparisons between age groups. There was no significance between macronutrients and gender, ethnicity, and locality.

### 3.3. Daily Micronutrient Intake According to Sex, Age, Ethnicity, and Locality

Table 3 shows the medians with interquartile ranges (IQR, 25–75) for daily intake of vitamins and minerals. Those over 70 years old had lower retinol intake than younger individuals (126.66 (7.37–317.97) vs. 310.18 (154.68–535.50) µg, *p* = 0.009); this was also the case for manganese (2.16 (1.45–2.94) vs. 2.93 (1.96–4.42) mg, *p* = 0.037), selenium (22.43 (14.56–32.40) vs. 40.23 (26.46–64.08) µg, *p* < 0.001), and zinc (4.18 (2.19–7.30) µg vs. 5.53 (4.29–8.16) µg, *p* = 0.031). Significant retinol intake differences were noted between those of FID/FOD and iTaukei (323.85 (105.97–419.08) µg vs. 154.32 (29.53–327.93) µg, *p* < 0.001); this was also the case for intake of thiamin (0.98 (0.63–1.46) mg vs. 0.68 (0.28–1.28) mg, *p* = 0.009), folate (212.77 (147.83–330.73) µg vs. 147.89 (92.90–304.60) µg, *p* = 0.032), iron (9.85 (6.92–13.37) mg vs. 7.35 (3.91–12.61) mg, *p* = 0.038), and manganese (2950.53 (2123.48–4516.93) µg vs. 2588.05 (1336.10–3895.72) µg, *p* = 0.021). Lower vitamin C intake was observed in Fijians of Indian/other (FID/FOD) descent relative to iTaukei: 38.93 (17.17–67.08) mg vs. 55.88 (24.83–113.38) mg, *p* = 0.012. Vitamin B_6_ median intake was higher in the rural areas relative to urban areas: 1.62 (0.15–2.96) vs. 1.20 (0.37–2.39) mg, *p* = 0.05. The median selenium intake was significantly inadequate across both age groups (22.43 (14.56–32.40) for those aged 55–69 vs. 40.23 (26.46–64.08) µg for Those > 70 years old, *p* < 0.01).

### 3.4. Percentage of Fijians Who Meet the Acceptable Macronutrient Distribution Range (AMDR) in Reference Values (NRVs) for Intake of Macro- and Micronutrients by Gender and Age

Nutrient adequacy analyses were based on the complete dietary intake dataset (n = 147). Regarding macronutrient intake, over half (60%) of the participants aged 55–69 met the recommended AMDR for carbohydrate intake (45–60% of total energy). In contrast, less than 30% met the AMDR for protein intake (15–25%), while approximately 20% exceeded the recommended AMDR for fat intake (20–35%) (Table 4). Among the participants > 70 years old or older, 50% of women met the AMDR for carbohydrate intake as compared to men (20%). Less than 30% met the AMDR for protein intake, and just over a quarter met the AMDR for fat intake (Table 4 and Table 5). Although mean macronutrient contributions (Table 2) fell within the recommended ranges for some nutrients, these findings indicate that a substantial proportion of the participants did not meet the recommended intake distributions.

Median micronutrient intakes (Table 3) provide an indication of central tendency; however, the prevalence of inadequacy is more appropriately reflected by the proportion of participants below recommended thresholds as reflected in Table 6 and Table 7. In relation to micronutrient intake, approximately 50% of the participants met the NRVs for vitamin B_6_, vitamin C, iron, phosphorus, and potassium for women only and not men. However, the burden of nutrient inadequacy was considerable, with vitamin E intake being notably insufficient in approximately 98–99% of older adults. High levels of inadequacy were also observed for vitamin B_12_ (61–88%), folate (76–88%), thiamine (60–88%), retinol (78–90%), calcium (88–100%), magnesium (83–94%), manganese (83–86%), selenium (74–100%), zinc (68–100%), and copper (62–88%). Potassium inadequacy was also substantial (88–90%), but it was observed among men only, highlighting a widespread nutritional deficit across age groups and, for potassium, a gender-specific pattern.

**Table 3:** Median daily micronutrient intake by sex, age, ethnicity, and locality (N = 147).

	All Participants N = 147	By Gender		By Age Groups		By Ethnicity		By Locality		
		Women (n = 78)	Men (n = 69)	55–69 Years (n = 129)	>70 Years (n = 18)	iTaukei (n = 61)	FID&FOD (n = 86)	Deuba (Rural) (n = 81)	Waidamudamu (Urban) (n = 66)	
Nutrients		Median (IQR 25–75)								*p*
Retinol (µg)	307.66 (134.98–494.53)	288.21 (93.28, 480.79)	309.39 (110.37–499.32)	310.18 (154.68–535.50)	126.66 (7.37–317.97)	154.32 (29.53–327.93)	323.85 (234.90–596.90)	268.67 (105.97–419.08)	319.21 (132.87–599.75)	0.009 ^†^ <0.001 ^‡^
Thiamin (mg)	0.86 (0.51–1.41)	0.78 (0.48–1.40)	0.90 (0.58–1.36)	0.87 (0.52–1.36)	0.76 (0.36–1.42)	0.68 (0.28–1.28)	0.98 (0.63–1.46)	0.76 (0.49–1.31)	0.92 (0.53–1.47)	0.009 ^‡^
Vitamin B_6_ (mg)	1.40 (0.52–2.86)	1.32 (0.51–2.87)	1.40 (0.45–2.86)	1.40 (0.51–2.85)	1.03 (0.34–2.96)	1.62 (0.50–2.90)	1.30 (0.48–2.78)	1.62 (0.65–2.96)	1.20 (0.37–2.39)	0.05 *
Vitamin B_12_ (µg)	1.23 (0.12–3.3)	0.97 (0.20–3.27)	1.14 (0.00–3.39)	1.34 (0.17–3.38)	0.83 (0.00–2.04)	1.13 (0.35–3.69)	1.11 (0.00–3.15)	1.74 (0.20–3.43)	0.83 (0.00–3.05)	
Folate (µg)	198.27 (112.42–326.42)	198.49 (102.52–317.82)	188.94 (119.89–324.91)	198.36 (117.01–323.37)	187.37 (88.94–268.96)	147.89 (92.90–304.60)	212.77 (147.83–330.73)	169.73 (103.57–284.30)	233.56 (119.27–340.62)	0.032 ^‡^
Vitamin C (mg)	42.15 (18.46–83.88)	49.24 (12.95–85.87)	40.36 (23.11–85.95)	42.16 (19.88–85.07)	47.84 (15.00–113.01)	55.88 (24.83–113.38)	38.93 (17.17–67.08)	42.13 (16.18–91.38)	46.50 (22.62–85.25)	0.012 ^‡^
Vitamin E (mg)	0.22 (0.00–2.01)	0.20 (0.00–1.56)	0.27 (0.00–2.21)	0.33 (0.00–2.12)	0.90 (0.00–1.29)	0.20 (0.00–0.91)	0.27 (0.00–2.75)	0.22 (0.00–1.27)	0.30 (0.00–2.95)	
Calcium (mg)	447.40 (273.55–828.64)	426.71 (254.00–805.98)	446.96 (317.45–836.17)	444.44 (280.24–817.52)	450.11 (170.48–825.67)	454.86 (174.92–798.49)	442.26 (328.13–831.89)	418.31 (258.96–837.48)	453.63 (303.75–803.16)_	
Iron (mg)	9.33 (5.61–12.74)	8.48 (5.36–12.59)	9.75 (5.78–13.23)	9.46 (6.30–12.67)	7.33 (2.92–14.78)	7.35 (3.91–12.61)	9.85 (6.92–13.37)	9.34 (5.64–12.81)	8.86 (4.92–13.69)	0.038 ^‡^
Magnesium (mg)	208.71 (137.39–274.86)	189.68 (134.53–271.25)	227.53 (160.32–276.93)	224.56 (154.42–273.10)	162.97 (80.37–329.38)	182.88 (129.62–266.82)	230.18 (154.62–277.64)	196.92 (142.02–275.16)	225.05 (139.02–278.57)	
Phosphorus (mg)	730.69 (485.45–1064.55)	701.25 (476.14–986.72)	791.01 (530.35–1110.76)	778.69 (522.79–1089.54)	642.02 (213.92–1029.17)	702.74 (455.67–991.00)	778.95 (517.85–1096.58)	752.38 (517.89–1101.79)	714.53 (484.66–1073.20)	
Potassium (mg)	1957.95 (1322.83–2664.47)	1864.17 (1237.79–2677.08)	2106.86 (1615.55–2654.00)	2049.93 (1456.87–2645.34)	1786.76 (946.17–3034.49)	1903.18 (1281.49–2650.60)	2050.42 (1440.23–2675.63)	1972.73 (1294.12–2634.85)	2021.10 (1413.03–2700.00)	
Manganese (mg)	2.84 (1.87–4.22)	2.71 (1.76–3.90)	2.95 (1.93–4.54)	2.93 (1.96–4.42)	2.16 (1.45–2.95)	2.58 (1.33–3.89)	2.95 (2.12–4.51)	2.89 (1.75–4.29)	2.71 (1.92–4.29)	0.037 ^†^ 0.021 ^‡^
Selenium (µg)	37.08 (24.41–54.15)	36.32 (22.90–50.05)	38.31 (25.40–64.08)	40.23 (26.46–64.08)	22.43 (14.56–32.40)	37.93 (22.73–71.99)	36.57 (25.34–53.10)	39.55 (25.75–59.30)	33.85 (21.70–53.24)	<0.001 ^†^
Copper (mg)	1.11 (0.75–1.60)	1.05 (0.70–1.51)	1.14 (0.82–1.70)	1.12 (0.83–1.60)	0.91 (0.49–1.60)	1.09 (0.65–1.47)	1.10 (0.87–1.61)	1.12 (0.75–1.74)	1.07 (0.74–1.51)	
Zinc (mg)	5.34 (3.84–7.72)	5.25 (3.62–7.38)	5.74 (4.25–8.26)	5.53 (4.29–8.16)	4.18 (2.19–7.30)	5.33 (3.41–7.38)	5.45 (4.29–8.21)	5.73 (3.87–8.18)	5.14 (3.62–7.72)	0.031 ^†^

^†^ Comparing all age groups, 55–69 years, and >70 years, *p* < 0.05, Mann–Whitney U univariate non-parametric test. ^‡^ Comparing all iTaukei and Fijians of Indian descent (FID) and Fijians of other descent (FOD), Mann–Whitney U univariate non-parametric test. * Comparing all localities, Deuba (rural) and Waidamudamu (urban), Mann–Whitney U univariate non-parametric test. *p*-values are shown only for variables reaching statistical significance (age, gender, ethnicity, and locality). There was no significance between micronutrients and gender.

**Table 4:** Proportion of Fijian older adults aged 55–69 years who met the nutrient reference values (NRVs) for Australia and New Zealand (reference category: 55–70 years) for macronutrient intake, stratified by gender and age.

NRV for Women 55–69 Years (n = 69)			NRV for Men 55–69 Years (n = 60)		
	Meeting NRVs n (%)	Not Meeting NRVs n (%)		Meeting NRVs n (%)	Not Meeting NRVs n (%)
Protein (EAR 37 g/d)	39 (57)	30 (43)	Protein (EAR 52 g/d)	22 (37)	38 (63)
Protein (EAR 0.60 g/kg per d)	32 (46)	37 (54)	Protein (EAR 0.68 g/kg per d)	29 (48)	31 (52)
%Energy from protein (15–25%)	15 (21)	54 (79)	%Energy from protein (15–25%)	12 (20)	48 (80)
%Energy from carbohydrate (45–65%)	46 (67)	22 (33)	%Energy from carbohydrate (45–65%)	46 (77)	14 (23)
%Energy from fat (20–35%)	25 (36)	44 (64)	%Energy from fat (20–35%)	28 (47)	32 (53)

**Table 5:** Proportion of Fijian older adults who met the nutrient reference values (NRVs) for Australia and New Zealand for intake of macronutrients by gender and age (>70 years).

NRV for Women > 70 Years (n = 9)			NRV for Men > 70 Years (n = 9)		
	Meeting NRVs n (%)	Not Meeting NRVs n (%)		Meeting NRVs n (%)	Not Meeting NRVs n (%)
Protein (EAR 46 g/d)	4 (44)	5 (56)	Protein (EAR 65 g/d)	0	9 (100)
Protein (EAR 0.75 g/kg per d)	0	9(100)	Protein (EAR 0.86 g/kg per d)	0	9 (100)
%Energy from protein (15–25%)	3 (33)	6 (67)	%Energy from protein (15–25%)	1 (12)	8 (88)
%Energy from CHO (45–65%)	6 (67)	3 (33)	%Energy from carbohydrate (45–65%)	2 (23)	7 (77)
%Energy from fat (20–35%)	2 (25)	7 (75)	%Energy from fat (20–35%)	2 (25)	7 (75)

**Note:** Nutrient adequacy analyses were based on participants with complete dietary intake data (n = 147). Analytical subgroups comprised women aged 55–69 years (n = 69), women aged >70 years (n = 9), men aged 55–69 years (n = 60), and men aged >70 years (n = 9). Percentages were calculated using the number of participants within each subgroup.

**Table 6:** Proportion of Fijian older adults who met the nutrient reference values (NRVs) for Australia and New Zealand for intake of micronutrients stratified by female gender and age group.

All Participants	Females 55–70 EAR			Females > 70 EAR			χ^2^
		Meeting EAR n (%)	Not Meeting EAR n (%)		Meeting EAR n(%)	Not Meeting EAR n (%)	
Nutrients							
*Vitamins*							
Vitamin B_6_ (mg)	1.3 mg/day	34 (50)	34 (50)	1.3 mg/day	12 (53)	11 (47)	0.384
Vitamin B_12_ (µg)	2.0 µg/day	21 (31)	48 (69)	2.0 µg/day	5 (24)	17 (76)	0.134
Vitamin C (mg)	30 mg/day	38 (56)	31 (44)	30 mg/day	11 (47)	12 (53)	0.862
Vitamin E (mg)	7 mg/day	1 (1)	68 (99)	7 mg/day	2 (10)	20 (90)	0.088
Folate (µg)	320 µg/day	14 (20)	55 (80)	320 µg/day	5 (20)	18 (80)	0.782
Thiamin (mg)	0.9 mg/day	26 (37)	43 (63)	0.9 mg/day	9 (40)	14 (60)	0.570
Retinol (µg)	500 µg/day	15 (22)	53 (78)	500 µg/day	2 (10)	20 (90)	0.127
*Minerals*							
Calcium (mg)	1100 mg/day	5 (8)	63 (92)	1100 mg/day	2 (8)	21 (92)	0.334
Iron (mg)	5 mg/day	49 (71)	20 (29)	5 mg/day	12 (54)	10 (46)	0.109
Magnesium (mg)	265 mg/day	14 (21)	54 (79)	265 mg/day	8 (34)	15 (66)	0.800
Manganese (mg)	5.0 mg/day	10 (14)	59 (86)	5.0 mg/day	4 (17)	19 (83)	0.881
Phosphorus (mg)	580 mg/day	37 (54)	32 (46)	580 mg/day	12 (51)	11 (49)	0.120
Potassium (mg)	2800 mg/day	58 (85)	11 (15)	2800 mg/day	17 (75)	6 (25)	0.272
Selenium (µg)	50 µg/day	18 (26)	51 (74)	50 µg/day	2 (7)	21 (93)	0.006
Zinc (mg)	6.5 mg/day	22 (32)	47 (68)	6.5 mg/day	6 (28)	16 (72)	0.302
Copper (mg)	1.2 mg/day	26 (37)	43 (62)	1.2 mg/day	7 (30)	16 (70)	0.143

χ^2^ Comparing micronutrients with all age groups, 55–69 years, and >70 years, *p* < 0.01. EAR = Estimated Average Requirement. This metric was used to estimate the likelihood of inadequate intake at the population level; meeting the EAR does not imply adequacy for individuals. Dietary intake data were evaluated against the Estimated Average Requirement (EAR). The EAR is defined as the daily intake level estimated to meet the requirements of 50% of individuals in a particular life stage and sex group. It is primarily used to assess nutrient adequacy at the population level, allowing estimation of the prevalence of inadequate intakes within a group. Intakes below the EAR indicate a higher likelihood of inadequacy. In this study, comparisons with the EAR were used to assess the likelihood of inadequate intake at the population level rather than to determine adequacy for individuals. Importantly, meeting or exceeding the EAR does not necessarily indicate sufficient intake for an individual, as it reflects only the median requirement.

**Table 7:** Proportion of Fijian older adults who met the nutrient reference values (NRVs) for Australia and New Zealand for intake of micronutrients stratified by male gender and age group.

	Males 55–69 EAR			Males > 70 EAR			χ^2^
All Participants		Meeting EAR n (%)	Not Meeting EAR n (%)		Meeting EAR n (%)	Not Meeting EAR n (%)	
Nutrients							
*Vitamins*							
Vitamin B_6_ (mg)	1.4 mg/day	30 (42)	42 (58)	1.4 mg/day	2 (25)	7 (75)	0.610
Vitamin B_12_ (µg)	2.0 µg/day	28 (39)	44 (61)	2.0 µg/day	1 (12)	8 (88)	0.134
Vitamin C (mg)	30 mg/day	40 (55)	33 (45)	30 mg/day	6 (62)	4 (38)	0.862
Vitamin E (mg)	10 mg/day	1 (2)	71 (98)	10 mg/day	0	9 (100)	0.230
Folate (µg)	320 µg/day	17 (24)	55 (76)	320 µg/day	1 (12)	8 (88)	0.782
Thiamin (mg)	1.0 mg/day	25 (35)	47 (65)	1.0 mg/day	1 (12)	8 (88)	0.583
Retinol (µg)	625 µg/day	13 (18)	59 (82)	625 µg/day	1 (12)	8 (88)	0.292
*Minerals*							
Calcium (mg)	840 mg/day	16 (22)	57 (88)	1100 mg/day	0	9 (100)	0.916 0.334
Iron (mg)	6 mg/day	47 (65)	25 (35)	6 mg/day	4 (48)	5 (52)	0.211
Magnesium (mg)	350 mg/day	5 (6)	68 (94)	350 mg/day	1 (12)	8 (88)	0.055
Manganese (mg)	5.5 mg/day	61 (84)	12 (16)	5.5 mg/day	8 (88)	1 (12)	0.881
Phosphorus (mg)	580 mg/day	48 (66)	25 (34)	580 mg/day	2 (25)	7 (75)	0.120
Potassium (mg)	3800 mg/day	7 (10)	66 (90)	3800 mg/day	1 (12)	8 (88)	0.707
Selenium (µg)	60 µg/day	18 (25)	55 (75)	60 µg/day	0	9 (100)	0.016
Zinc (mg)	12.0 mg/day	6 (8)	67 (92)	12.0 mg/day	0	9 (100)	0.319
Copper (mg)	1.7 mg/day	16 (22)	57 (78)	1.7 mg/day	1 (12)	8 (88)	0.609

χ^2^ Comparing micronutrients with all age groups, 55–69 years, and >70 years, *p* < 0.01. EAR = Estimated Average Requirement. This metric is used to estimate the likelihood of inadequate intake at the population level; meeting the EAR does not imply adequacy for individuals. Dietary intake data were evaluated against the Estimated Average Requirement (EAR). The EAR is defined as the daily intake level estimated to meet the requirements of 50% of individuals in a particular life stage and sex group. It is primarily used to assess nutrient adequacy at the population level, allowing the prevalence of inadequate intakes within a group to be estimated. Intakes below the EAR indicate a higher likelihood of inadequacy. In this study, comparisons with the EAR were used to assess the likelihood of inadequate intake at the population level rather than to determine adequacy for individuals. Importantly, meeting or exceeding the EAR does not necessarily indicate sufficient intake for an individual, as it reflects only the median requirement.

### 3.5. Contribution of Food Sources to Energy and Macronutrient Intakes

Supplementary File, Figure S1a–d, presents an overview of the food sources of macronutrients and energy intake. The key dietary sources of energy were bread and baked products (34%); mixed cooked dishes (16%); and fruits and vegetables, nuts, and legumes (12%), collectively accounting for 60% of energy intake. Cereal and grain products; non-alcoholic beverages; meat, poultry, and meat alternatives; and sugar, honey, and related products accounted for a further 25% of energy intake.

The key dietary sources of carbohydrates were bread and baked products (38%); fruits and vegetables, nuts, and legumes (18%); and cereal and grain products (16%), accounting for 60% of intake. Sources of total sugars were sugars, honey, and related products (8%); non-alcoholic beverages (9%); and mixed cooked dishes (7%), accounting for 24% of carbohydrate intake. The dietary sources of protein were bread and baked products (27%), mixed cooked dishes (19%), and meat, poultry, and meat alternatives (16%), accounting for 60% intake. Seafood and seafood products accounted for 5% of protein intake, while egg and egg products and dairy accounted for less than 4% each of protein intake.

The key dietary sources of fat were mixed cooked dishes (33%), bread and bakery products (29%), and ‘meat, poultry, and meat alternatives’ (9%), accounting for 70% of intake. Edible oils, oil emulsions, and non-alcoholic beverages each accounted for a further 5% of fat intake.

For food sources of sugars, sugars, honey, and related products contributed the most (26.5%), along with non-alcoholic beverages (26%), followed by bread and bakery products (20%), mixed cooked dishes (10.4%), and fruits and vegetables, nuts, and legumes (9%). Of the other sources of total sugars, dairy accounted for 3%; cereal and grain products made up 1.4%; sauces, dressings, spreads, and dips accounted for 1.3%; and less than 0.5% came from convenience foods, coconut products, seafoods and seafood products, eggs and egg products, and edible oil and oil emulsions. See Supplementary File, Figure S2.

### 3.6. Contribution of Food Sources to Micronutrient Intake

Supplementary File, Figure S3a presents an overview of food sources of micronutrients of concern in older adults. The food sources of folate were primarily non-alcoholic beverages (24%); mixed cooked dishes (19%); bread and bakery products; fruits, vegetables, nuts, and legumes (15%); and cereal and grain products (14%). Dietary intake from eggs and egg products, dairy, and convenience foods made up less than 5% of the food source contributions.

The main dietary sources of calcium were non-alcoholic beverages (29%), bread and bakery products (27%), and mixed cooked dishes (16%). Less than 10% of calcium intake was from fruits, vegetables, nuts, and legumes; dairy; sugars, honey, and related products; and cereal and grain products. See Supplementary File, Figure S3b.

The dietary sources of retinol were mixed cooked dishes (26%), bread and bakery products (25%), non-alcoholic beverages (11%), and meat, poultry, and meat alternatives. Fruits and vegetables, nuts and legumes, edible oil and oil emulsions, egg and egg products, seafood and seafood products, and dairy contributed less than 10% each to retinol intake. See Supplementary File, Figure S4a.

The main dietary sources of vitamin B_12_ were meat, poultry, and meat alternatives (31%), followed by non-alcoholic beverages (26%), seafood and seafood products (15%), and mixed cooked dishes (13%). Eggs and egg products, bread and bakery products, and dairy together made up less than 10% of the Vitamin B12 contribution, with very small contributions from other food sources. See Supplementary File, Figure S4b.

The main dietary sources of selenium were bread and bakery products (21%), followed by seafood and seafood products and mixed cooked dishes, each accounting for 19% of total selenium intake. Less than 10% of selenium intake was contributed by fruits, vegetables, nuts, and legumes (9.8%); meat, poultry, and meat alternatives; egg and egg products; and cereal and grain products. See Supplementary File, Figure S4c.

The main dietary sources of zinc were mixed cooked dishes (18%); bread and bakery products (17%); meat, poultry, and meat alternatives (16%); cereal and grain products (15%); and fruits, vegetables, nuts, and legumes (13%). Other food sources contributed less than 10% of total zinc intake. See Supplementary File, Figure S4d.

## 4. Discussion

This study is the first to provide a detailed examination of energy, nutrient intake, and food sources of nutrients among older adults aged 55 and above in Fiji. Existing national surveys provide limited age-specific dietary data for this population. The 2025 WHO STEPwise approach to Noncommunicable Disease (NCD) Risk Factor Surveillance, commonly known as the STEPS survey, reported only the frequency of fruit and vegetable consumption and salt and sugar intake amongst adults aged 18–69 [3], while the 2015 NNS reported nutrient intake for adults aged 18 and above without a clearly defined upper age limit [11]. Consequently, in the absence of specific dietary intake and food source data for Fijian adults aged 55 and above, findings from studies conducted in New Zealand and Australia, alongside the NHMRC dietary guidelines [10], were used to interpret the results of this study. Overall, the findings indicate that some older people have lower-than-recommended total energy intakes, with adequate proportions of energy and nutrients derived from fat and carbohydrates but relatively lower contributions of energy from protein. Inadequate intakes of several key micronutrients were also observed among the participants based on reported values.

We observed that the mean daily energy intakes for men and women were 6.9 MJ and 6.3 MJ, respectively, and lower among those aged ≥70 years (5.3 MJ). These values describe overall intake levels; however, mean estimates do not capture the distribution of intake within the population. When interpreted alongside the proportion of participants with low BMI values (54.2% < 24 kg/m^2^), these findings suggest that a substantial proportion of individuals may have inadequate energy intakes. With no existing data on older-adult dietary intake in Fiji and the Pacific with which to compare our findings, we found that older Fijians’ energy intakes are lower than the energy intakes for older adults in the 2008/09 New Zealand Adult Nutrition Survey (NZANS), where the median daily energy intake for men and women was 8.4 MJ and 6.4 MJ in the 65–74-year age group and 7.4 MJ and 5.5 MJ for those aged 75 years and older [36].

The estimated lower energy intake we have reported may be due to underreporting of total energy intake that could have derived from other energy sources—such as alcohol and kava (*Piper methysticum*)—not considered in our study [37] or previous studies, including snack foods [26,28]. It may also reflect age-related appetite decline [38,39], economic or food access constraints [40,41], or cultural dietary patterns characterised by lower energy density [42,43]. Furthermore, the low dietary intakes from this study indicate a potential risk of malnutrition, especially in relation to protein and essential micronutrients, which may contribute to sarcopenia and frailty. These findings underscore the need for targeted public health strategies to support adequate energy consumption in Fiji’s ageing population [38,44]. As more than half of the participants (54.2%) had a BMI less than 24 kg/m^2^, which is now considered the cut-off for underweight for Those > 65 years old [34,35], we applied BMI cut-points intended for adults > 65 to a broader ≥ 55-year population, the majority of whom were aged 55–69. This action may have resulted in misclassification and potential overestimation of underweight prevalence. Nonetheless, the relatively low reported energy intakes suggest potential underreporting, a known limitation of self-reported dietary assessments. As EI:BMR was not assessed, the extent of underreporting cannot be quantified; however, values below 1.2 are typically indicative of underreporting and should be considered when interpreting the findings.

The older Fijians in this study did not meet the AMDR for protein (15–25%), with only a small proportion of the participants falling within the recommended range. Although the mean protein contribution to total energy intake was 13%, this value alone does not reflect adequacy at the population level. Prevalence-based analysis demonstrated that most of the participants did not meet the recommended protein intake distributions [10]. In contrast, mean carbohydrate (56%) and fat (28%) contributions fell within AMDRs; however, only 50–60% of participants met the recommended carbohydrate range, and an even smaller proportion met protein recommendations. This shows that mean values may mask substantial variability in intake and should be interpreted alongside distribution-based measures.

Adults ≥ 70 years old obtained an even greater proportion of energy from carbohydrates (63%), a shift that may further dilute protein density given age-related declines in appetite and energy intake. Older New Zealanders and Australians generally report higher protein contributions, typically 15–16% in New Zealand and about 19% in Australia, and slightly higher fat but lower carbohydrate proportions than those observed in older Fijians [36,42]. These differences suggest that older Fijians have comparatively lower protein density and a more carbohydrate-heavy macronutrient profile. In addition, this study did not account for socioeconomic factors or living arrangements, which may influence dietary intake. Previous research suggests that individuals with lower income may consume a higher proportion of carbohydrate-rich foods [43]. Therefore, the findings should be interpreted in consideration of these unmeasured factors.

The mean protein intake for all participants observed in this study (51.07 g/day) was substantially lower than that reported in the 2015 Fiji National Nutrition Survey (149 g/day). This marked difference likely reflects several important factors. First, the study populations are not directly comparable, as the NNS included adults aged ≥18 years, whereas this study focused exclusively on older adults ≥ 55 years old. Older adults typically have lower total energy and protein intakes due to age-related physiological changes, including reduced appetite and altered metabolic demands. Second, differences in dietary assessment methods, portion size estimation, and data collection procedures between the two studies may have contributed to variability in reported intake. Third, underreporting of dietary intake particularly among older populations may also partly explain the lower estimates observed in this study. These factors highlight the need for caution when directly comparing the two datasets.

Our findings show that both men and women in this study consumed a mean daily protein intake of 0.68 g/kg of bodyweight. While this approximates the EAR (0.60–0.68 g/kg/day), adequacy cannot be inferred from mean intake alone. Evaluation based on recommended thresholds indicated that a considerable proportion of the participants did not achieve protein intakes consistent with optimal functional targets (≥1.0–1.2 g/kg/day). These findings suggest that, although average intake may appear adequate for deficiency prevention, intake is likely insufficient for optimal health outcomes in a large proportion of individuals [10,12,13,45]. Fijian participants had a lower protein intake adjusted for bodyweight relative to previous findings among older community-dwelling adults aged 65–74 in New Zealand, where the median daily protein intake was 1.16 g/kg for men and 1.09 g/kg for women [22]. Shortfalls in protein intake may be associated with lower muscle mass, strength, and physical performance, which are key mediators of fall risk, while higher, well-distributed protein supports muscle protein synthesis (MPS), preserves lean mass, and maintains strength and recovery from catabolic stressors [46,47]. Our data indicate that although the participants met the minimum requirements, protein intake was not optimised for healthy ageing. Elevating protein intakes consistent with contemporary guidance may be required to yield clinically meaningful benefits for muscle health, function, and fall risk among older Fijians.

In this study, older Fijians showed widespread inadequacies in key micronutrients. The median intakes for several nutrients (excluding Vitamin B6) fell below EAR values, suggesting potential population-level inadequacy; however, the extent of risk is more accurately reflected by the proportion of participants not meeting recommended intakes. More than half of the participants consumed below the EAR for thiamine, folate, and vitamin C, while vitamin B_6_ requirements were met by approximately half of the participants. The median intakes of manganese, selenium, and zinc were also below recommended levels, and prevalence-based analysis confirmed there was substantial inadequacy across multiple micronutrients. When stratified by locality, vitamin B_6_ intake was significantly higher in rural participants than in urban ones (median: 1.62 vs. 1.20 mg/day, *p* = 0.05). This locality difference may reflect variations in dietary patterns, food availability, or socioeconomic factors, including higher poverty rates between rural and urban areas [25]. For example, differences in access to locally produced foods or reliance on market-based food systems may contribute to this pattern. However, these findings should be interpreted with caution, as we did not directly assess the underlying determinants of locality-based dietary variation to determine the prevalence of inadequacy.

The median intakes of manganese, selenium, and zinc were also below recommended levels, indicating broader insufficiencies across multiple micronutrient pathways. Calcium intake was notably low, with the median intake of 447 mg/day falling well below the EAR (840 mg/day). Approximately half of the participants did not meet recommended intake levels, indicating a substantial risk of inadequacy [10]. These findings, combined with high sodium intake and a high prevalence of hypertension (71.6%), suggest potential compounding effects on bone and cardiovascular health [28,48].

The micronutrient insufficiencies among older Fijians in this study align with national surveillance data. The Fiji NNS 2015 similarly reported suboptimal intakes of retinol and calcium, with over half of adults falling short of the recommended dietary intake (RDI) for both nutrients [11]. National data from a survey in 2004 also highlighted co-existing deficiencies in iron, folate, and vitamin A, particularly among Indian women, suggesting long-standing population-level risks [49]. Comparisons with regional data reflect parallel trends. In the National Nutrition Survey, older New Zealanders were found to consume inadequate amounts of calcium and showed low retinol levels; this was especially true among Pacific adults [36]. Australian findings echo this pattern, with intakes among older adults frequently falling below recommendations for calcium and zinc [42]. Consistent with the micronutrient inadequacies observed in this study, low intakes of folate, Vitamin E, Ca, Mg, Se, and Zn have been observed, as reported in the diets of community-living older adults [50] and residents of aged care facilities in New Zealand [51]. Collectively, these regional comparisons stress that micronutrient insufficiency remains a persistent issue among older adults in Pacific populations, including Fijians. Enhancing dietary micronutrient adequacy may support immune function, cognitive health, and bone and metabolic wellbeing [52] while also helping to reduce the risk of multimorbidity among older adults [53].

Our findings should be interpreted within the context of Fiji’s evolving nutrition landscape. Older adults are increasingly exposed to processed, high-sodium foods while facing inconsistent access to nutrient-dense options, contributing to both micronutrient inadequacies and rising non-communicable disease prevalence [26]. Evidence from narrative reviews emphasises the ongoing gaps in dietary quality and the need for a strengthened policy focus on nutrition in later life [54]. Overall, micronutrient intake among older Fijians, particularly iTaukei adults and those ≥70 years old, is substantially lower than Australian and New Zealand EARs and reflects long-term nutritional vulnerabilities documented in national surveys [11,24,49]. The observed patterns underscore the urgent need for targeted, culturally appropriate interventions to improve micronutrient status and support healthy ageing in Fiji.

The dietary patterns of older Fijians show that bread and bakery products are major sources of macronutrient intake regarding protein, carbohydrates, fat, and total energy, followed by mixed cooked dishes and meat or meat alternatives, while fruits, vegetables, nuts, and legumes made modest contributions. The Fiji NNS 2015 identified fish and seafood as the leading protein source [11], whereas our study shows that processed staples and bakery items are the major sources, aligning with recent findings indicating that ultra-processed foods (UPFs), particularly breads, baked goods, and sugar-sweetened beverages, now contribute substantially to energy, sugar, sodium, and fat intake [26,28]. New Zealand data similarly indicate that older adults rely heavily on bread, milk, meat, and seafood for protein, with bread also being the main source of carbohydrates and energy and butter, margarine, and baked goods providing most dietary fat [36,55].

This study’s findings indicate that individuals of FID/FOD reported higher fat and lower carbohydrate intakes than iTaukei, whereas iTaukei patterns were more carbohydrate-heavy, consistent with detailed dietary recording studies showing Indo-Fijians’ greater reliance on rice and flour products and the emphasis among iTaukei on traditionally grown starchy root crops such as cassava, dalo, yams, and flour-based items [11,24,56,57]. FID/FOD cooking preparations differ in the use of oil and spices for seasoning, while iTaukei prefer traditional boiling, stewing, grilling, and baking as cooking methods, which may affect sensory and food preferences and food intake [58]. Within this context, feasible dietary improvements include increasing the quantity of culturally familiar protein sources such as fish, eggs, dairy products, and legumes; substituting bread-based meals with traditional starchy roots; and enhancing vegetable content within mixed dishes. These tailored modifications align with existing eating patterns and may meaningfully improve nutrient density among older adults.

Older-adult micronutrient intakes in this study were derived from mixed cooked dishes, bread and bakery products, and non-alcoholic beverages, suggesting a strong reliance on composite or processed foods rather than individual nutrient-dense sources. This pattern differs from that described in Fiji NNS 2015, which identified more traditional and whole-food sources as the primary contributors to key micronutrients. For example, while the NNS reported that calcium was obtained from fish and seafood (26.6%) and green leafy vegetables (23.8%), we found that calcium was predominantly obtained from non-alcoholic beverages and bakery products, with minimal intake via dairy or leafy vegetables. Similarly, in the NNS, vitamin A was found to be obtained from vegetables (44.3%) and animal-source foods (22%), whereas in our study, vitamin A was found to be obtained from mixed cooked dishes and bakery foods. The 2015 NNS [11] reported that thiamin and zinc intakes were heavily driven by cereal-based foods and seafood, respectively, yet, in this study, these nutrients were distributed across mixed dishes, bakery products, cereals, and plant-based foods rather than being concentrated in traditional staples or seafood. Calcium intake in older adults can be improved via consumption of dairy or plant-based milk (in liquid or powdered form); these products can also be added to soups or stews (gravies) as thickeners. Older adults can also consume tinned fish with soft bones (sardines), fresh fish, and local green leafy vegetables such as *bele* and *rourou*. Fortified foods such as flour, cereals, or plant-based milk can also help, where available and affordable.

Given Fiji’s high and rising NCD burden, including obesity, diabetes, and hypertension among adults, the dietary patterns observed for older adults ≥ 55 and above with low protein intake, prevalent micronutrient inadequacies, and heavy reliance on bread, bakery items, and SSBs represent controllable levers for policy and practice [59]. Structural measures that reshape food environments (reducing UPF and SSB availability and marketing, reformulating salt/sugar/fat in popular bread and bakery products, etc.) are likely to yield population-level benefits, particularly when coupled with initiatives that improve the affordability of and access to nutrient-dense, especially protein-rich, local foods [6,60]. For clinicians and community health teams, these findings justify strengthening existing surveillance data by ensuring older adults are represented in sufficient numbers in national nutrition surveys and the STEPS survey. Priority should also be given to improving protein adequacy from 13% of energy to the upper AMDR and from 0.68 g/kg/day to ≥1.0–1.2 g/kg/day [10,12,13,46]. (We observed that energy intakes (EIs) among older Fijian adults in this study were lower than the EERs outlined in the Australia/New Zealand NRV, compromising overall micronutrient adequacy, as reflected in the consistently low intakes of key micronutrients. As EI decreases with age, obtaining an adequate micronutrient intake presents a challenge, especially when recommendations are higher for older adults.

### Strengths and Limitations

A key strength of this study is that it provides baseline, detailed dietary intake data for older adults in Fiji’s Central Division, a population for which evidence has been lacking. The use of standardised 24 h MPR dietary assessment methods and inclusion of both macronutrient and micronutrient analyses deepen our understanding of diet quality in this age group. The Intake24 application was a useful technological tool in this study. Additionally, the study captures ethnic and age-related differences, offering insights for culturally tailored interventions.

However, several limitations should be acknowledged. Firstly, this study’s cross-sectional design precludes causal inference and limits the ability to assess temporal changes in dietary patterns. Secondly, the sample was drawn from one geographic division, potentially restricting generalizability to other regions of Fiji with different food environments and cultural practices. Thirdly, dietary intake was obtained through self-reporting, which is subject to recall bias and potential under- or overestimation of energy and nutrient intake. Fourthly, key nutrients important for older adults were missing, such as dietary fibre, beta carotene, cholesterol, and vitamin B_12_, and some beverage and food sources, such as alcohol, kava, and snack foods, were not recorded; therefore, corresponding analyses were not conducted. A further limitation is our use of the 24 h dietary recall assessment method. We adapted Intake24 to the Fijian context by matching food composition data to common foods/recipes in Fiji based on macronutrient composition, thereby limiting interpretation of micronutrient intake and micronutrient intake findings, which should therefore be treated as explorative in this paper. In addition, the use of a 24 h dietary recall and not repeated measures introduced limitations regarding assessing both macro- and micronutrient intake, as it captures only short-term consumption and may be affected by recall bias and daily variability. Moreover, differences in dietary assessment methods across published studies may partly explain some of the discrepancies observed in the reported nutrient intakes. Finally, while nutrient adequacy was assessed against reference values, biochemical measures of micronutrient status were not collected; these would have strengthened the interpretation of deficiencies. Additionally, future research should incorporate socioeconomic and household-level variables to better explain their influence on dietary patterns.

## 5. Conclusions

The dietary profile of community-dwelling older adults aged 55 years and older living in the central Division of Fiji was found to be characterised by low energy intake, a low contribution of protein to total energy, and broad micronutrient inadequacies. Notably, vitamin E intake was critically low, with approximately 98–99% of participants failing to meet recommended levels, alongside a high prevalence of inadequacy across several other essential micronutrients. Dietary patterns were dominated by high reliance on bread and bakery items, mixed cooked dishes, and substantial intake of non-alcoholic beverages such as sugar-sweetened beverages. These patterns are consistent with the Pacific’s nutrition transition and Fiji’s growing diet-related non-communicable disease burden [29,54,61,62].

This study provides the first evidence-based data with which to inform targeted nutrition interventions and policies for older adults in Fiji. Strategies for reversing the transition towards more processed and energy-dense dietary patterns need to be considered together with interventions that increase energy intake, promote consumption of protein-rich local foods, improve micronutrient intake, and support healthier dietary behaviours to promote healthy ageing.

## Data Availability

A data-sharing agreement was obtained from the University of New South Wales, The George Institute (TGI) collaborators, who are custodians of the data and co-authors of this paper.

## Supplementary Materials

The following supporting information can be downloaded at: https://www.mdpi.com/article/10.3390/nu18142354/s1, Table S1: Units of measurement and Nutrient reference values for energy intake and micronutrients; Table S2: 15 Food categories and corresponding food sources; Figure S1: Contribution of Food Sources to Macronutrient and Energy Intake: (a) energy, (b) fat, (c) protein, (d) carbohydrate in older adults in the Central division, Fiji; Figure S2: Contribution of Food Sources to total sugars in the Central division, Fiji; Figure S3: Contribution of Food Sources to Micronutrients (a) Folate, (b) Calcium in older adults in the Central division, Fiji; Figure S4: Contribution of Food Sources to Micronutrients (a) Retinol, (b) Vitamin B12, (c) Seleni-um and (d) Zinc in older adults in the Central division, Fiji.

## Abbreviations

The following abbreviations are used in this manuscript:

NCDs	Non-communicable diseases
LE	Life expectancy
NFHGs	National Food and Health Guidelines
SPC	Secretariat of Pacific Communities
BW	Bodyweight
EAR	Estimated average requirement
BMR	Basal metabolic rate
TEE	Total energy expenditure
NNS	National Nutrition Survey
24 h MPR	24 h multi-pass recall
GACD	Global Alliance for Chronic Diseases
FNU	Fiji National University
HHREC	Human Health Research Ethics Committee
MUHEC	Massey University Human Ethics Committee
FID/FOD	Fijians of Indian descent/Fijians of other descent
BMI	Body mass index
WHO	World Health Organization
SFAs	Saturated fatty acids
NRV	Nutrient reference value
AMDR	Acceptable macronutrient distribution range
SD	Standard deviation
CI	Confidence interval
NHMRC	National Health and Medical Research Council
MJ/d	Megajoules per day
EER	Estimated energy requirement
IQR	Interquartile range
STEPS	STEPwise approach to NCD Risk Factor Surveillance Survey
NZANS	New Zealand Adult Nutrition Survey
MPS	Muscle protein synthesis
RDI	Recommended dietary intake
EI	Energy intake
