# Supplementary material for: Dietary Intake Among Community-Dwelling Adults Aged 55 Years and Older in the Central Division, Fiji
Source: Nutrients. 2026 Jul 17;18(14):2354. doi: 10.3390/nu18142354 (PMC13415802; doi:10.3390/nu18142354)
Supplement: Supplementary file 1 [file nutrients-18-02354-s001.zip › nutrients-4327087-supplementary.pdf]

Supplementary Tables and Figures

Table S1 Units of measurement and Nutrient reference values for energy intake and micronutrients

Nutrients	Units/day	Nutrient Reference Value(NRV): EAR (Estimated average requirement)			
		Men		Women	
		51-69 years	>70 years	51-69 years	>70 years
Energy	MJ	9.3-11.7	8.3-10.8	8.3-9.6	7.4-9.2
Vitamin C	mg	30	30	30	30
Calcium	mg	840	1,100	1,100	1,100
Iron	mg	6.0	6.0	5	5
Copper	mg	1.6	1.6	1.2	1.2
Folate	µg	320	320	320	320
Magnesium	mg	350	350	265	265
Manganese	µg	5.5	5.5	5	5
Phosphorus	mg	580	580	580	580
Potassium	mg	3,800	3,800	2800	2800
Retinol	µg	625	625	500	500
Selenium	µg	60	60	50	50
Thiamin	mg	1.0	1.0	0.9	0.9
Vitamin B12	µg	2.0	2.0	2.0	2.0
Vitamin B6	mg	1.4	1.4	1.3	1.3
Vitamin E	mg	10	10	7.0	7.0
Zinc	mg	12	12	6.2	6.2

Table S2 15 Food categories and corresponding food sources

	Food categories	Food sources
1	Bread and bakery products	Bread, pastries, roti, chapati, puri, babakau(fry bread), buns in coconut cream, scones Pikelet, donuts
2	Cereal and grain products	Breakfast cereals, oats, noodles
3	Coconut products	Coconut cream ,milk
4	Convenience foods	Takeaway foods, pre-packed salads, and sand-wiches
5	Dairy	Milk products(liquid and powder), cheese, yogurt
6	Edible oils and emulsions	Oils, fats
7	Egg and egg products	Egg and egg products
8	Fruit, vegetables, nuts and legumes	All fruits, green leafy vegetables, colored vegeta-bles, seeds, nuts and pulses, legumes, lentils
9	Meat, poultry and meat alternatives	Include sausages, offal, canned meats
10	Mixed cooked dishes	All mixed cooked dishes containing protein and vegetables or vegetarian
11	Non-alcoholic beverages	Milo, coffee, tea,Ovaltine, Cocoa Instant powder juice(Tangs), lemon grass, lemon leaves, including sugar sweetened beverages(SSBs)
12	Sauces, dressings, spreads and dips	Tomato sauce, mayonnaise, soy sauce, butter, mar-garine, all dips
13	Seafood and seafood products	Seafood, shellfish, crustaceans, canned tuna, tin fish, sardines
14	Snack food(sweet and savory snacks)	Sweets-homemade Indian sweets, packaged chips, savory
15	Table sugars, honey and related products (such as syrups and molasses)	Refined sugars, honey, jam, syrups, molasses

Figure S1

Contribution of Food Sources to Macronutrient and Energy Intake: (a) energy, (b) fat, (c) protein, (d) carbohydrate in older adults in the Central division, Fiji.

a) Energy

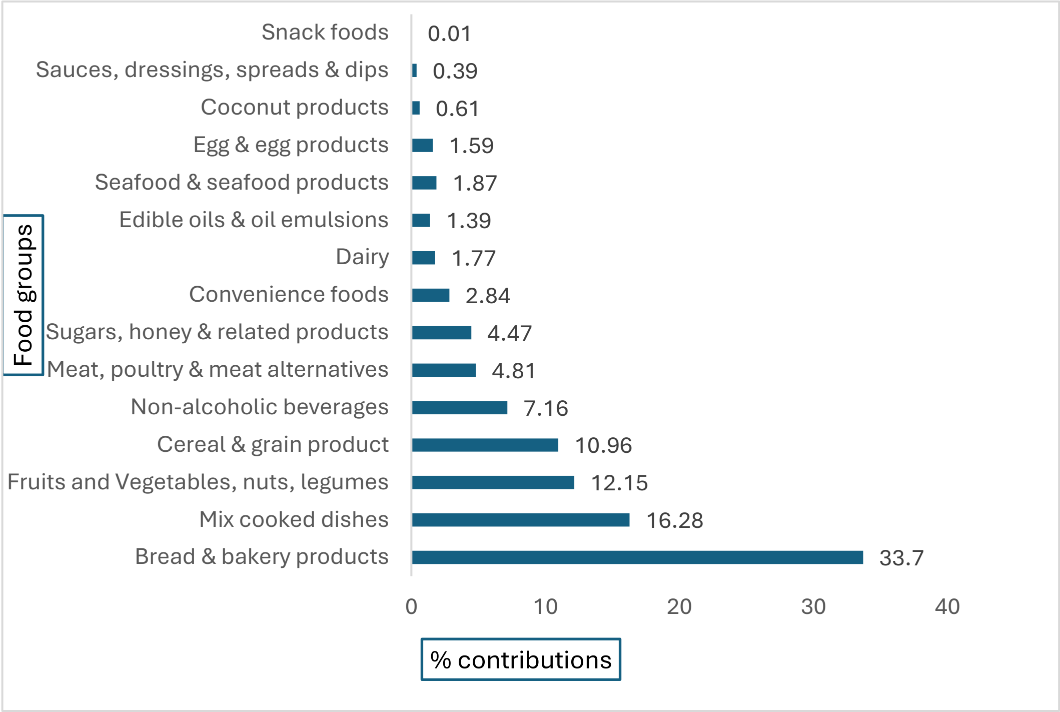

b) Fat

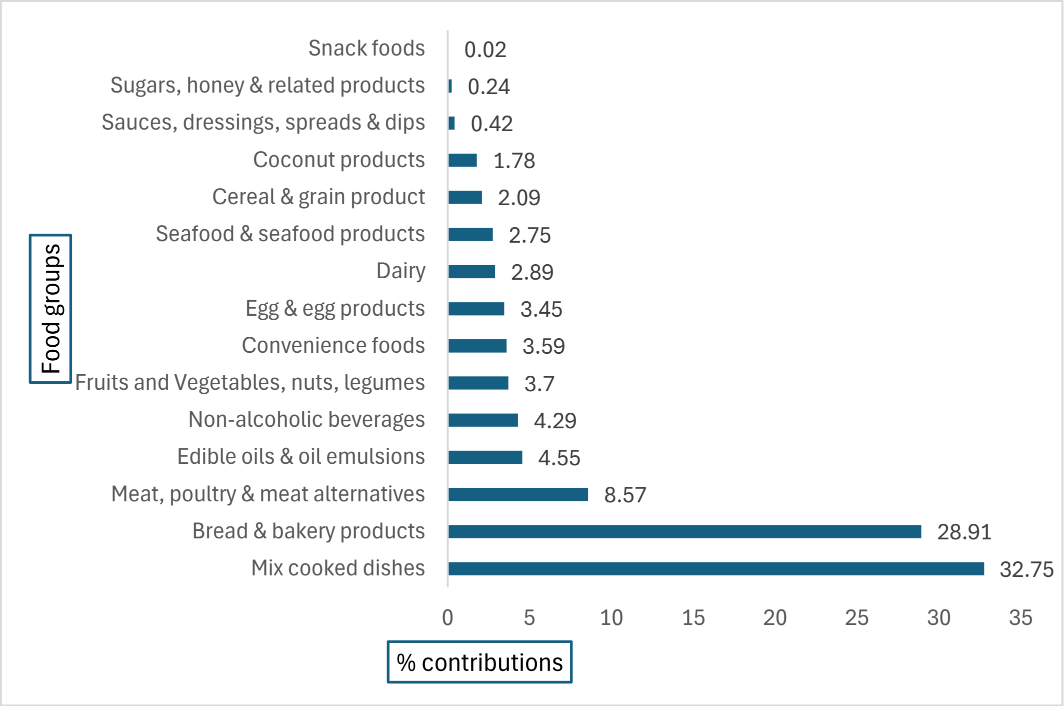

c) Protein

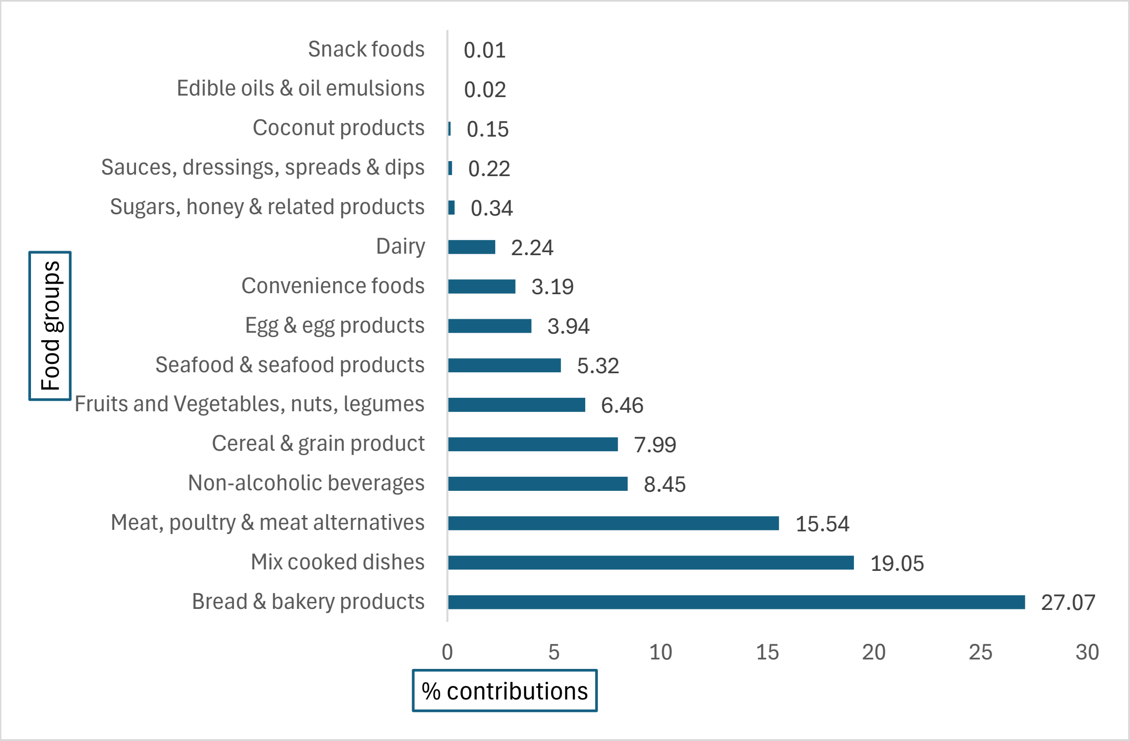

d) Carbohydrates

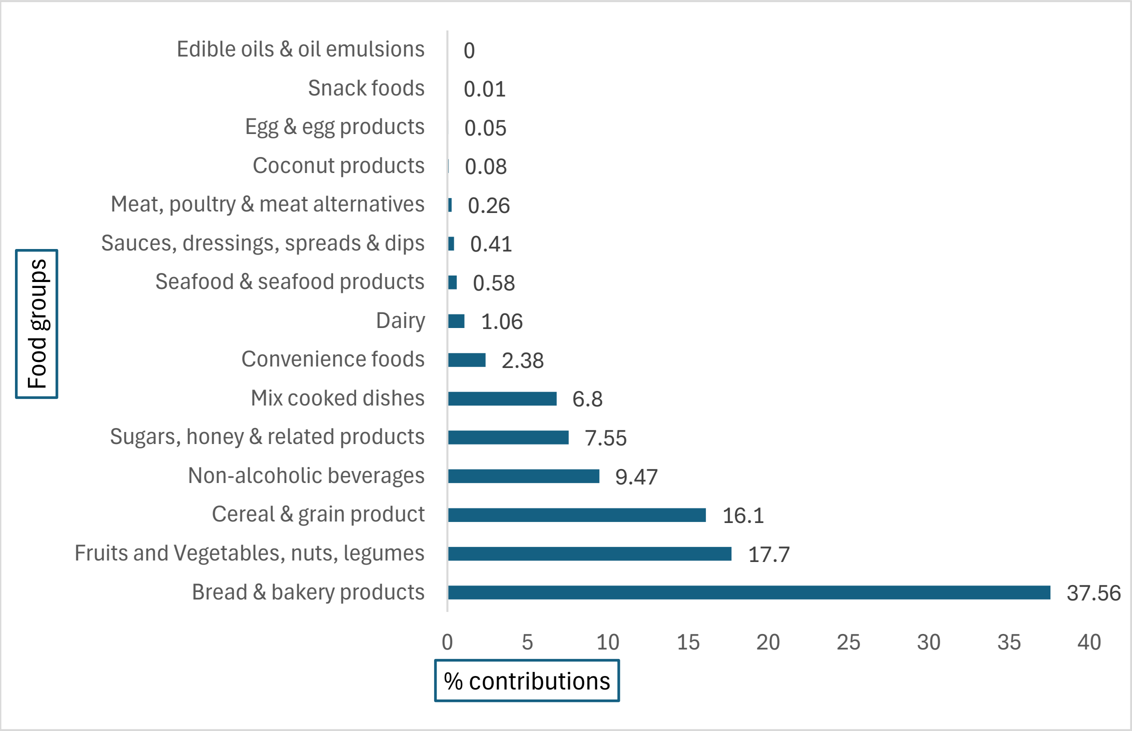

Figure S2: Contribution of Food Sources to total sugars in the Central division, Fiji.

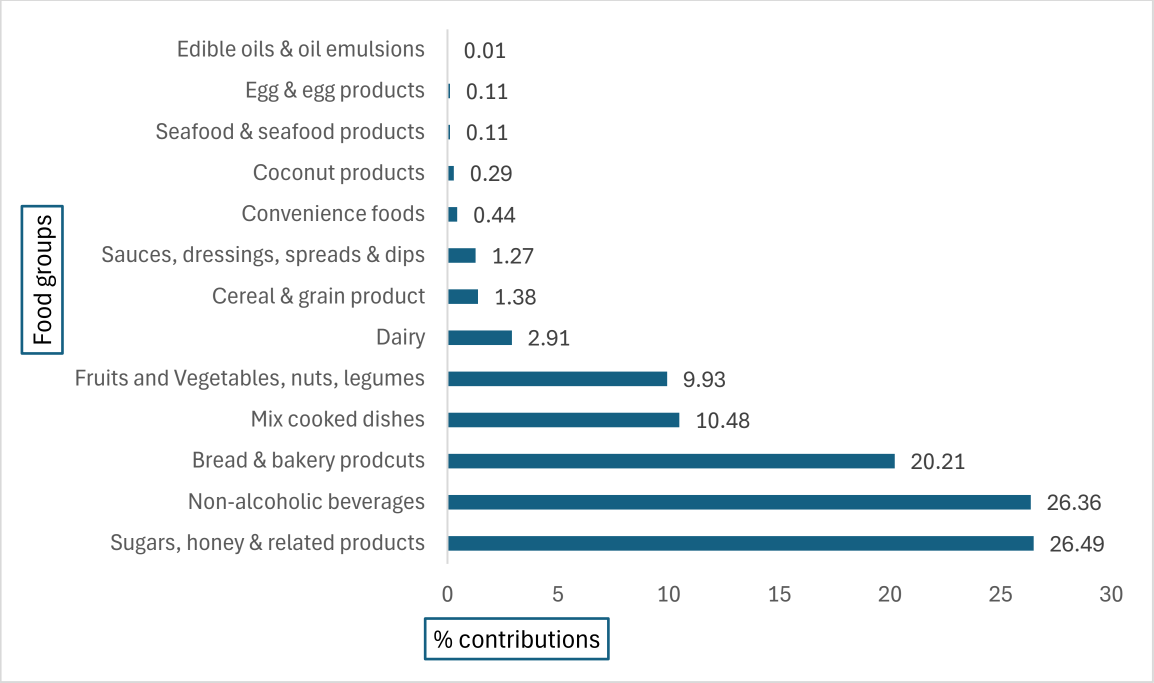

Figure S3: Contribution of Food Sources to Micronutrients a) Folate, b) Calcium in older adults in the Central division, Fiji.

a) Folate

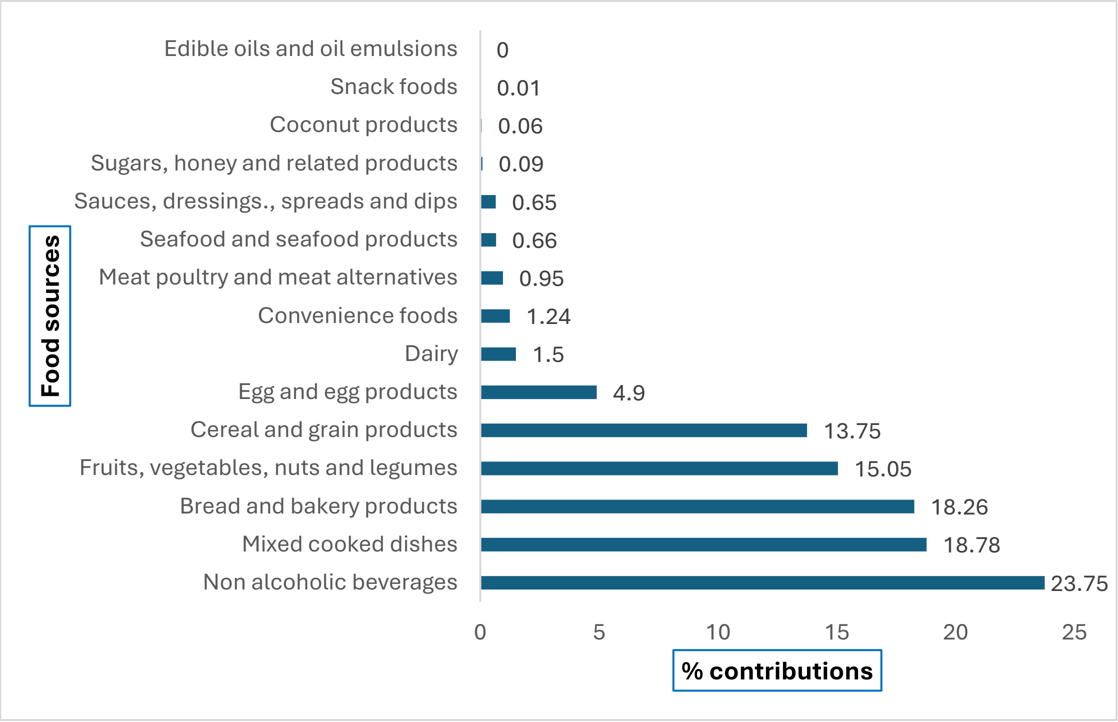

b) Calcium

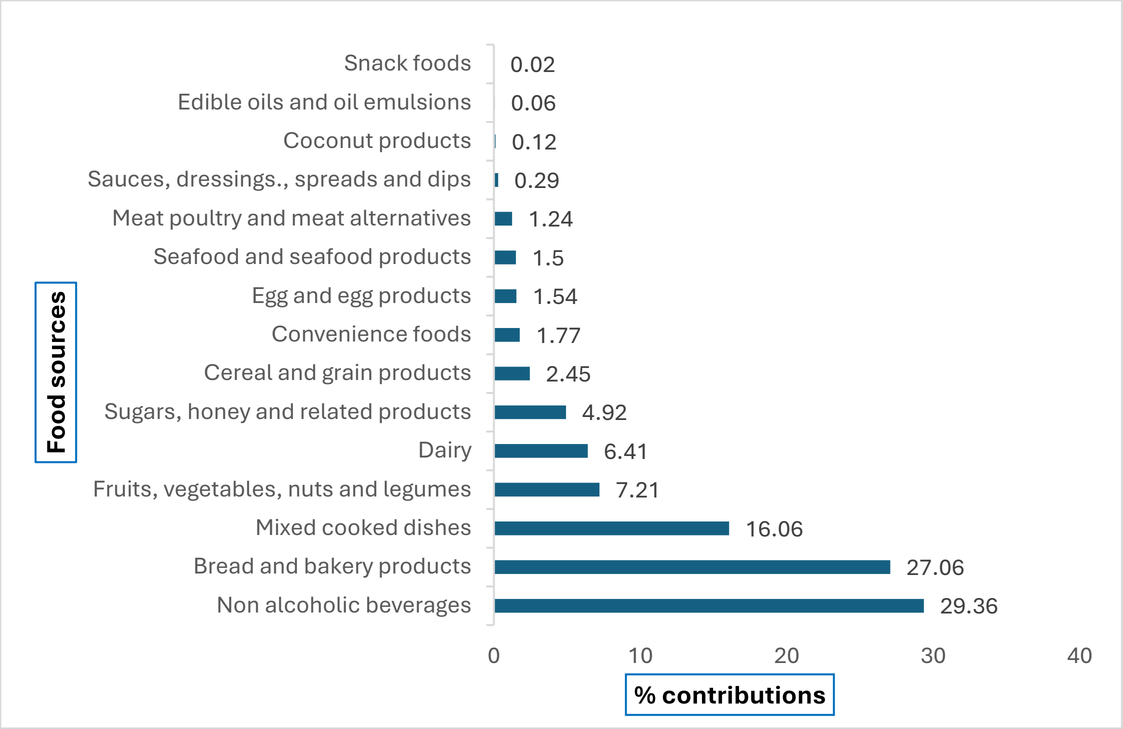

Figure S4: Contribution of Food Sources to Micronutrients (a) Retinol, (b) Vitamin B_{12}, (c) Selenium and (d) Zinc in older adults in the Central division, Fiji.

a) Retinol

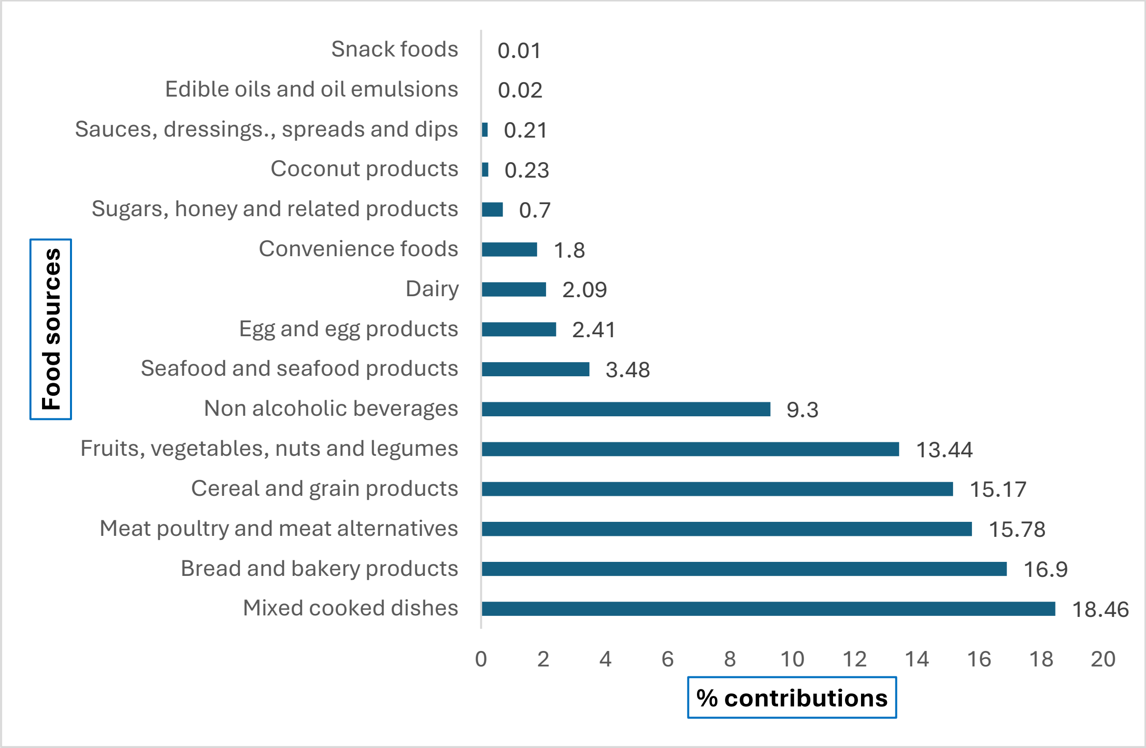

b) Vitamin B_{12}

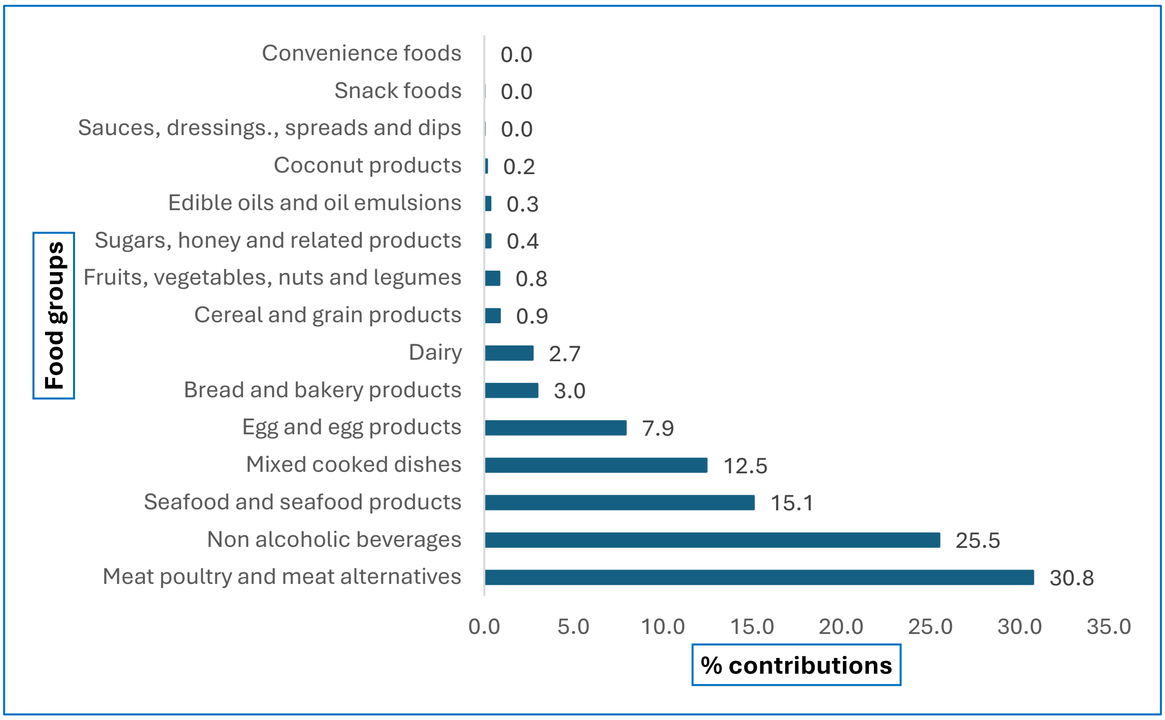

c) Selenium

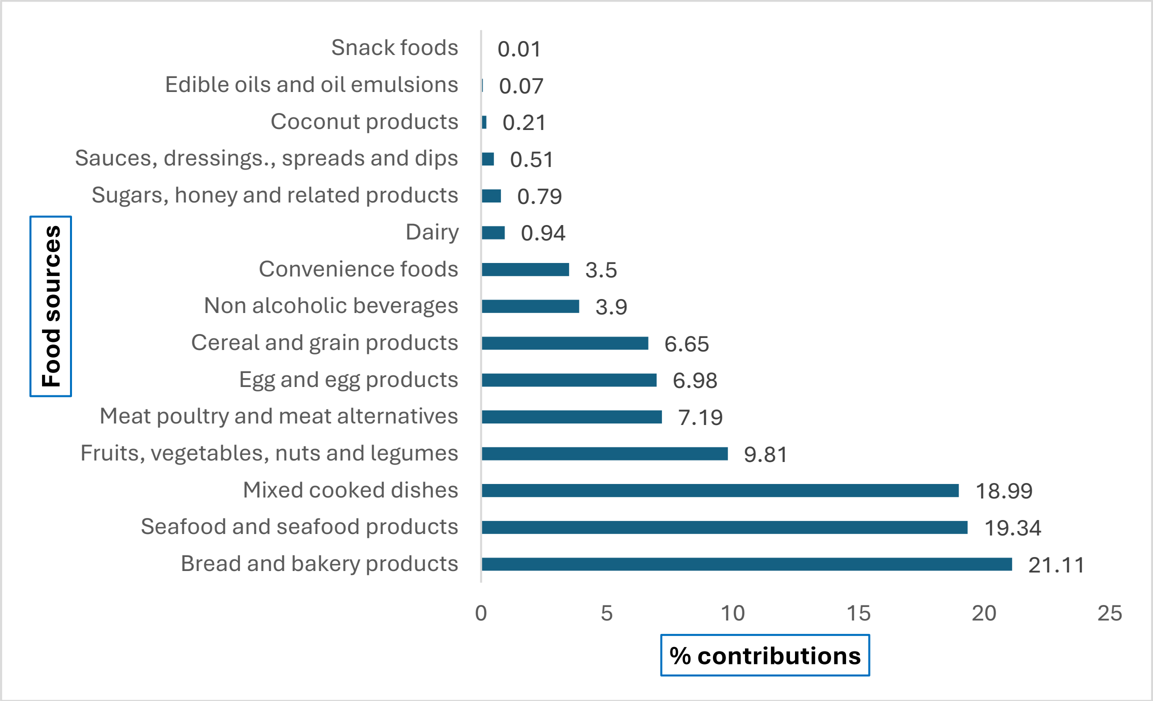

d) Zinc

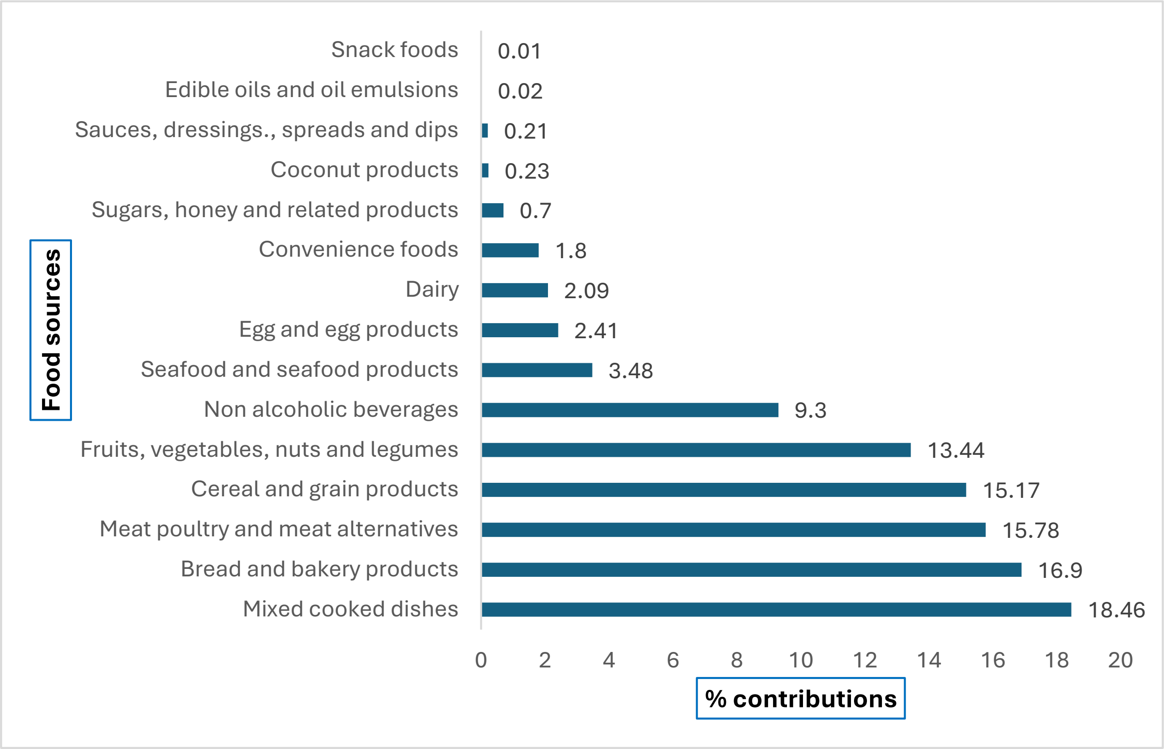
Snack foods
Edible oils and oil emulsions
Sauces, dressings., spreads and dips
Coconut products
Sugars, honey and related products
Convenience foods
Dairy
Egg and egg products
Seafood and seafood products
Non alcoholic beverages
Fruits, vegetables, nuts and legumes
Cereal and grain products
Meat poultry and meat alternatives
Bread and bakery products
Mixed cooked dishes
0.01
0.02
0.21
0.23
0.7
1.8
2.09
2.41
3.48
9.3
13.44
15.17
15.78
16.9
18.46
Food sources
0
2
4
6
8
10
12
14
16
18
20
% contributions
